# Bias‐Triggered Conductivity Relaxation (BCR): A Unique Tool to Simultaneously Investigate Thermodynamics, Kinetics, and Electrostatic Effects of Oxygen Reactions in MIEC Thin Films

**DOI:** 10.1002/adma.73869

**Published:** 2026-06-30

**Authors:** Alexander Stangl, Alexander Schmid, Adeel Riaz, Jürgen Fleig, Arnaud Badel

**Affiliations:** ^1^ Atominstitut TU Wien Vienna Austria; ^2^ CNRS Grenoble INP Institut Néel Université Grenoble Alpes Grenoble France; ^3^ CNRS Grenoble INP LMGP Université Grenoble Alpes Grenoble France; ^4^ Institute of Chemical Technologies and Analytics TU Wien Vienna Austria; ^5^ CNRS Grenoble INP G2ELab Institut Néel Université Grenoble Alpes Grenoble France

**Keywords:** electrostatic surface potential, functional oxides, in situ methodology, mixed ionic electronic conductors, oxygen reaction kinetics, surface defects

## Abstract

Mixed ionic‐electronic transfer (MIET) reactions, such as the oxygen reduction reaction (ORR) at oxide surfaces, are of paramount importance to manifold technologically highly relevant processes, and fundamental understanding must be developed to improve performance and tailor highly efficient electrodes and catalysts. Understanding such complex multi‐step reactions requires the study of kinetic processes, underlying thermodynamic properties, i.e., ionic and electronic defect concentrations, and electrostatic surface effects. However, conventional techniques struggle to uncover the complete picture within the same sample/measurement. Here, we overcome this limitation by introducing bias‐triggered conductivity relaxation (BCR) as a novel tool to investigate MIET reactions on oxides. It is based on alternating out‐of‐plane coulometric titration/polarization and in‐plane electrical conductivity relaxation measurements, providing simultaneous electronic, ionic, and extraordinarily rich surface kinetics information. This innovative combination of electrical and chemical driving forces synergizes information depth, with enhanced time resolution, versatility, and speed, yet it lifts the weaknesses of the individual approaches, while remaining cost‐effective and surprisingly simple. Furthermore, BCR allows to disentangle overpotential induced electrostatic modifications of the surface kinetics in a unique manner. We showcase the advantages of BCR in this work by studying the ORR in model (La, Sr)FeO_3‐δ_ thin film electrodes and reporting on their thermodynamic and kinetic properties.

## Graphical abstract

This work develops bias‐triggered conductivity relaxation as a novel technique to study oxygen reactions in mixed ionic‐electronic conducting thin films by integrating electrochemical titration and electrical conductivity relaxation to achieve synchronous multi‐parameter characterization, providing simultaneous electronic, ionic, and extraordinarily rich surface kinetics information in a unique manner.

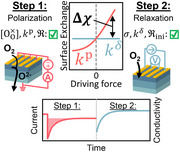

## 1 Introduction

From fusion enabling superconductors [1] to renewable energy storage solutions based on highly efficient solid oxide fuel cells and batteries [2, 3], mixed ionic electronic conducting (MIEC) oxide materials are central to various emerging technologies to foster the energy transition and combat anthropogenic climate change [4]. In this context, oxygen transport reactions are twofold important. First, functional properties of oxides are highly influenced by their oxygen stoichiometry, making the precise control of the oxygen content key for superior materials performance [5, 6], while on the other hand, the operation principle of various energy‐related electrochemical processes is based on the oxygen reduction reaction (ORR) itself [7]. The accelerated development of tuned metal oxides relies on innovative approaches to materials synthesis and nano‐engineering [8], the improved understanding of ion kinetics and point defects [9, 10], and on a constantly expanding experimental toolbox [11].

The ORR describes the incorporation of a gaseous oxygen molecule, O_2,gas_, into two bulk crystal sites. For an oxygen vacancy‐based defect chemistry, it can be written as:

(1) $$O_{2,\mathrm{gas}}+2v_{O}^{••}\;\to 2O_{O}^{\times }+4h^{•}$$

with the oxygen vacancy, $v_{O}^{••}$, the electronic hole, $h^{•}$, and the filled bulk oxygen site, $O_{O}^{\times }$. In contrast to its apparent simplicity, the complexity of the ORR cannot be overstated, as the arrow in Equation (1) covers a succession of elementary reaction steps, including adsorption, ionic, and electronic charge transfer reactions across gas–solid electrochemical interfaces, dissociation, recombination with crystal sites, and various diffusion processes in the presence of mixed electrostatic and chemical gradients [12]. These elementary mechanisms may occur several times in an a priori unknown order, while it is generally considered that one of these fundamental reaction mechanisms is limiting the overall reaction rate, thus being the rate‐determining step (RDS). Identifying this rate‐limiting reaction of the ORR is an essential step toward materials‐by‐design with enhanced electrochemical performance, durability, and possibly improved sustainability due to reduced critical raw materials consumption. Countless efforts have been undertaken to shed light on the RDS [13, 14, 15, 16, 17, 18]. Thin film electrodes are especially suitable for mechanistic studies, as gas and bulk diffusion contributions are reduced, and they can be manufactured with well‐defined geometries, microstructures, compositions, etc, which also benefits their implementation into applications. Given the apparent difficulties to follow these mixed ionic electronic transfer (MIET) reactions at the atomic and sub‐atomic level [19], macroscopic approaches, based on the analysis of pressure dependencies, have crystallized to be the most promising ones [12]. Oxygen transport processes are frequently studied using electrochemical impedance spectroscopy (EIS) [20], isotope tracer profiling using secondary ion mass spectroscopy (SIMS) [21], and recently developed in situ Raman spectroscopy approaches [22, 23, 24], relaxation experiments, such as electrical conductivity relaxation [25], or in situ XRD [26], as well as dynamic equilibrium approaches, including electrochemical titration and current density‐overpotential (*j* − η) measurements [27], which provide complementary information on the inquired system. A general rate equation for the rate‐determining step was formulated in ref. [28] and can be written in the limit of low coverage as:

(2) $$\begin{array}{c} R=\vec{R}-\overset{↼}{R}\\ \vec{R}\propto \underset{\mathrm{Adsorbates}}{\underset{︸}{{\left[{\mathrm{pO}}_{2}\right]}^{\frac{1}{n}}}}\underset{\mathrm{Defe}\mathrm{cts}}{\underset{︸}{\prod_{i}{\left[i\right]}^{\nu_{i}}}}\times \underset{\begin{array}{c} \mathrm{Elec}\mathrm{tros}\mathrm{tatic}\\ \mathrm{term} \end{array}}{\underset{︸}{e^{\frac{\vec{\beta }e\chi }{k_{B}T}}}}\times \underset{\begin{array}{c} \mathrm{Over}\mathrm{pote}\mathrm{ntial}\\ \mathrm{effe}\mathrm{cts} \end{array}}{\underset{︸}{e^{\frac{\vec{\beta^{\prime }}e\Delta \chi \left(\eta \right)}{k_{B}T}}}}\\ \vec{R}\propto \underset{\mathrm{Defe}\mathrm{cts}}{\underset{︸}{\prod_{j}{\left[j\right]}^{\nu_{j}}}}\times \underset{\begin{array}{c} \mathrm{Elec}\mathrm{tros}\mathrm{tatic}\\ \mathrm{term} \end{array}}{\underset{︸}{e^{\frac{\overset{↼}{\beta }e\chi }{k_{B}T}}}}\times \underset{\begin{array}{c} \mathrm{Over}\mathrm{pote}\mathrm{ntial}\\ \mathrm{effe}\mathrm{cts} \end{array}}{\underset{︸}{e^{\frac{\overset{↼}{\beta^{\prime }}e\Delta \chi \left(\eta \right)}{k_{B}T}}}} \end{array}$$

The forward and backward rates, $\vec{ℜ}$ and $\overset{↼}{ℜ}$ constitute of contributions from adsorbates, ionic, and electronic defect species, *i* and *j*, with their respective reaction orders *v_i_* and *v_j_*, respectively, as well as electrostatic effects, due to the presence of a surface potential step, χ, and its variation under an applied overpotential, Δχ(η). The latter term subsumes electrostatic effects arising with the application of an overpotential, such as changes of the surface dipole layer, e.g., by a modified coverage of charged adsorbate species, which is also reflected in changes of the work function [29, 30, 31]. Defects can appear directly in the reaction rate if they participate in the RDS or indirectly through intermediate states (i.e. via the equilibrium constant, *K*_eq_). Note that defect concentrations and consequently *K*_eq_ depend as well on the overpotential. Sophisticated measurement designs, as developed for example by Merkle [32], Schmid [33], and Guan [34] for chemical and electrical experiments, allow to separate and eliminate of certain defect contributions in Equation (2). Nevertheless, elucidating the ORR reaction pathway requires access to both kinetic and thermodynamic materials parameters, including defect concentrations as well as reaction rates, exchange coefficients, and their variations under current. Up to now, simultaneous determination of all these properties was not feasible, despite advanced approaches based on the integration of complementary techniques [35, 36, 37]. Thus, the mechanistic interpretation of kinetic processes is frequently based on different assumptions (e.g., Brouwer approximation), (bulk) literature data, and/or several experiments under different conditions with samples subject to evolution, and may therefore not be sufficiently accurate for the specific investigated material (e.g., thin films).

Here, we aim to tackle this issue by combining, in an alternating operation mode, out‐of‐plane electrochemical titration and current‐overpotential measurements with in‐plane electrical conductivity relaxation (ECR) measurements to create the bias‐triggered conductivity relaxation (BCR) technique. This approach is simple and easy to implement, yet allows for obtaining all relevant information within a single experiment, including the ionic and electronic defect state of MIEC oxides, as well as kinetic parameters, such as the surface exchange coefficient and reaction rates. Furthermore, limitations of the individual techniques (such as flush time, accessible *p*O_2_ range, and inherent electrostatic modification of the surface) are lifted, while their information depths are synergistically combined. This provides enriched and novel insights and the unmatched opportunity to analyze bias‐induced, electrostatic modifications of the oxygen surface reaction rates by directly comparing chemical and electrical experiments. The full potential and the substantial advantages of this new technique to study various versatile MIEC materials are demonstrated in the following by studying (La,Sr)FeO_3‐δ_ thin film electrodes.

## 2 Methods

### 2.1 Key Concepts of Known *j* − η and ECR Measurements

**FIGURE 1:**
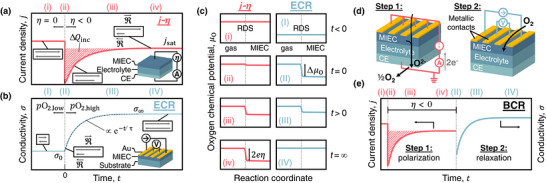
Overview of the measurement principle for (a) current density—overpotential (*j* − η) and (b) electrical conductivity relaxation (ECR) measurements with sketches of the sample configurations. The different stages in (a, b) are labeled with majuscule and minuscule Roman numerals, respectively: (i & I) initial equilibrium, (ii & II) perturbation of equilibrium via application of η or jump in *p* **O**_2_, (iii & III) relaxation toward the new equilibrium/steady state (iv & IV). Gray arrows in (a,b) illustrate forward and backward reaction rates. The MIEC undergoes a stoichiometric change, quantifiable via the marked red area in (a). The oxygen chemical potential profiles across the surface of the MIEC for the different stages of *j* − η and ECR measurements (i–iv and I–IV) are drafted in (c). (d) Special device architecture enables two different cell configurations. Fast switching between the electrode configurations is enabled via a digital relay. (e) Principle of novel bias‐triggered conductivity relaxation (BCR) technique based on the combination of polarization (step 1) and relaxation (step 2) measurements.

Time‐dependent current density‐overpotential (*j* − η) measurements (which include coulometric titration) and electrical conductivity relaxation (ECR) are known standard approaches for the investigation of oxygen kinetics and defects in MIEC materials. Their basic measurement principles are summarized in Figure 1 and in the following, while Table S1 in the Note S2 provides an overview of the accessible kinetic and thermodynamic parameters for each technique.

Insight into reaction kinetics is typically obtained via an intentionally introduced (small) distortion of the equilibrium, e.g. in the case of current density‐overpotential/coulometric titration measurements via the application of an electrical potential across an electrochemical cell. That is a pure ionic‐conducting electrolyte sandwiched between a MIEC material of interest as the working electrode (WE) and a highly active counter electrode (CE), as illustrated in Figure 1a. If the surface reactions of the WE are slow compared to the oxygen transport properties of the remaining device components (including oxygen diffusion in the WE), a DC voltage applied across the cell translates (partially) into an overpotential, η, which modifies the oxygen chemical potential (and thereby the oxygen stoichiometry) in the entire WE according to:

(3) $$\mu_{O,\mathrm{WE}}=\mu_{O,\mathrm{CE}}\;\left(pO_{2,\mathrm{ref}}\right)+2e\eta$$

with µ_O,CE_(*p*O_2,ref_) denoting the chemical potential of oxygen at the CE (being in equilibrium with the oxygen partial pressure of the surrounding gas phase *p*O_2,ref_). The oxygen stoichiometry of the MIEC is changed by pumping oxygen ions from/into the MIEC via the electrochemical cell, which resembles the building up of a cell voltage in a battery during charging. The time evolution of the µ_O_ across the gas‐MIEC interface is drafted in Figure 1c (left panels). The four stages correspond to: (i) the MIEC is in equilibrium with the atmosphere prior to the application of a voltage (*t* < 0). (ii) A negative bias results in a drop of µ_O_ over the RDS, which (iii) rapidly increases with time until (iv) the steady state is reached with Δ µ_O_ =  2eη. The new oxygen chemical potential inside the MIEC can be related to an effective oxygen partial pressure, *p*O_2,eff_ (assuming ideal gas behavior) using the Nernst equation:

(4) $$p\;O_{2,\mathrm{eff}}=pO_{2,\mathrm{ref}}e^{\frac{4F\eta }{RT}}$$

with *F* and *R* being the Faraday and gas constants, respectively. Essentially, as a second‐order effect of the applied voltage, the jump in µ_O_ drives a continuous net oxygen flux across the surface, corresponding to a leakage current. Therefore, despite being an electrical experiment, there is a true chemical potential gradient involved across the WE surface and consequently the saturation current density, *j*_sat_(η), in the steady state, is a measure of the net surface reaction rate, ℜ:

(5) $$\frac{j_{\mathrm{sat}}\left(\eta \right)}{2e}=ℜ=\vec{ℜ}\;-\overset{↼}{ℜ}$$

As this current consumes a fraction of the applied voltage, the attainable overpotential in the WE reduces to:

(6) $$\eta =U_{\mathrm{DC}}\;-j_{\mathrm{sat}}A_{\mathrm{film}}R_{\mathrm{CE}+\mathrm{EL}}$$

with the surface area of the sample, *A*_film_, and the summed resistances, *R*_CE + EL_, of all cell components (including ionic diffusion through the substrate, contact resistances, counter electrode, and possible small interfacial resistances, etc.), but the working electrode. From the flown charge, i.e., from the time‐dependence of the current, we can extract defect chemical thermodynamic information as for usual coulometric titration studies. Assuming a constant oxygen reaction rate at the surface and thus a constant leakage flux throughout the entire titration process, i.e. *j*_leak_ (*t*) = *j*_sat_ , we can quantify the relative change in oxygen off‐stoichiometry, Δδ, of the WE via the area under the *j*(*t*) curve (marked red in Figure 1a):

(7) $$\Delta \delta =\frac{V_{\mathrm{uc}}}{2e\cdot d_{\mathrm{film}}}\;\int \left(j\left(t\right)-j_{\mathrm{sat}}\right)\mathrm{dt}$$

with the sample thickness, *d*_film_, and the unit cell volume *V*_uc_. Thus, we get information as for pure coulometric titration, i.e. with negligible leakage current. More realistically, the leakage current density is not constant but increases gradually with µ_O,WE_ (and thus with the cell voltage). Hence, Δδ as given in Equation (7) acts more as a lower bound, while its uncertainty increases with the magnitude of *j*_sat_ (relative to *j*(*t*)) and the step size of η. However, for a rate‐limiting surface step being much more resistive than all other kinetic processes in the cell (reaction at the CE, ion transport in the electrolyte, charge transfer into the MIEC, transport in the MIEC) and thus $\eta \approx U_{\mathrm{DC}}$, the correction for *j*_sat_ in Equation (5) is small (a few %), as shown in the Note S5, and meaningful non‐stoichiometry data can be obtained despite a non‐constant dc current flow.

A major drawback of polarization measurements is the inherent, very non‐trivial coupling of the applied bias with charged surface defects, the electrostatic surface potential, and the Fermi level of the bulk (and thus the work function). This intrinsically causes a modification of the reaction rate of the RDS and severely complicates the mechanistic interpretation of MIET reactions at gas–solid interfaces.

In conventional ECR experiments, on the other hand, kinetic processes are triggered directly via a jump in the oxygen partial pressure surrounding the sample, while monitoring the conductivity, σ, of the material. Resulting conductivity relaxation curves can be analyzed using solutions of Fick's diffusion model to obtain the chemical surface exchange coefficient, *k* ^δ^, and/or the chemical diffusion coefficient, *D* ^δ^ [38]. Accurate determination of transport parameters, however, requires that the gas exchange of the atmosphere is much faster than the kinetics of the sample, to avoid interference of these two processes [39]. Ideally, the *p*O_2_ vs. time curve follows a square wave (i.e. instantaneous partial pressure jump), which is experimentally difficult to achieve and limits its applicability to temperatures where kinetics are sufficiently slow.

An exemplary oxidation step is shown in Figure 1b: (I) in the initial state at low *p*O_2_, forward and backward reaction rates are in equilibrium (gray arrows). (II) With the rise of the *p*O_2_, $\vec{ℜ}$ jumps to the new value (due to its direct dependence on *p*O_2_), while (III) $\overset{↼}{ℜ}$ only increases gradually. This leads to a net flux of oxygen, *J*(*t*) (equal to the net reaction rate, ℜ(*t*)), into the MIEC, which vanishes once the new equilibrium state is reached (IV). Within a first‐order, linear kinetic regime, the net oxygen flux through the surface is proportional to the oxygen concentration difference between the first surface layer, *c*(*t*) and the value corresponding to equilibrium with the gas phase (*c*_∞_):

(8) $$J\;\left(t\right)=k^{\delta }\;\left(c_{\infty }-c\left(t\right)\;\right)$$

with the proportionality constant being *k* ^δ^. For a surface‐limited thin film within the plane‐sheet approximation and a linear relation between σ and *c*, the normalised conductivity transient can be modeled using a single exponential function:

(9) $$\sigma_{\mathrm{norm}}=\frac{\sigma \left(t\right)-\sigma_{0}}{\sigma_{\infty }-\sigma_{0}}=1-e^{-\frac{t}{\tau }}$$

with the time constant, τ  = *d*_film_/*k* ^δ^ . The evolution of the µ_O_ profile is drawn in Figure 1c (right panels). Prior to a forward jump, the initial, low µ_O_ is constant across the surface. In the ideal case, at *t*  =  0 a step in µ_O_ builds up instantaneously with the step size Δ µ_O_ =  *RT*/2log (*p*O_2,low_/*p*O_2,high_). With increasing time, Δµ_O_(*t*) decreases, as the new equilibrium is approached. For a surface‐limited reaction, µ_O_ is at all times homogeneous within the full MIEC bulk with no gradient along the thickness.

Both methods lead to kinetic information on the oxygen exchange process at the WE surface, namely *j*_sat_ in *j* − η studies and *k* ^δ^ in ECR measurements. Interestingly, the measurement times required to get these complementary surface kinetic data are generally rather different. This is due to the fact that in time‐dependent *j* − η studies, the chemical potential changes in the MIEC take place via an “outsourcing” of the ORR to the much faster CE. Hence, all the (smaller) resistances except the rate‐limiting surface step are decisive, while for ECR the rate‐determining surface step limits the equilibration process, which thus becomes slower. In other words, the chemical capacitance of the WE gets charged via the (generally small) *R*_CE + EL_ in *j* − η measurements and discharged via the (larger) *R*_WE_ in ECR experiments. Moreover, the analysis of the time‐dependent current in *j* − η measurements allow us to quantify the defect chemical state of the MIEC, which is otherwise challenging to obtain, while the ECR method provides insight into the material's electronic state.

### 2.2 Novel Bias‐Triggered Conductivity Relaxation (BCR)

Here, we propose to merge current density‐overpotential measurements (including coulometric titration) and electrical conductivity relaxation into a single methodology, bias‐triggered conductivity relaxation (BCR), to synergize their individual merits and overcome their inherent weaknesses. This novel combination ultimately provides highly efficient access to all relevant parameters for the in‐depth characterization of MIET reactions in oxides and opens up many novel considerations. In comparison to reported joint titration‐conductivity approaches, relaxation, and titration measurements are directly performed on the same specimen [40], the kinetic dimension is analyzed rather than suppressed [41, 42, 43, 44, 45], and electrochemical experiments are matched against chemical ones [37], which significantly enriches our results and simplifies setup and sample preparation.

For BCR measurements, we deploy a specific electrochemical cell architecture with four metallic top electrodes, as shown in Figure 1d, and to our knowledge originally introduced by Yugami et al. [44]. In a first step, the four metallic top electrodes are short‐circuited to give a surface equipotential (as indicated in Figure 1d), and the sample is biased across the electrolyte until the current saturates (10–100 s) to establish an effective *p*O_2_ inside the MIEC. This is equivalent to conventional *j* − η measurements, see Figure 1e. This step is followed by a rapid, digital relay‐controlled switch of the electrode configuration to cut the out‐of‐plane connection and contact the top electrodes individually for in‐plane, four‐point conductivity measurements. Upon removal of the overpotential, the effective *p*O_2_ can no longer be retained within the MIEC, and the system relaxes back to the thermodynamic equilibrium state defined by the *p*O_2,ref_. This relaxation is monitored without further delay by electrical conductivity measurements using small excitation currents. Very short switching and fast recording times (10–100 ms) allow the capture of even very fast kinetic processes. Cutting the external out‐of‐plane connection ensures that any ionic flow through the electrolyte is stalled, and relaxation takes place exclusively via the native MIEC top surface.

Compared to standard ECR measurements, the step in the *p*O_2_ of the gas phase is replaced with a virtually instantaneous electrochemical step in *p*O_2,eff_, whereas the initial and final states are defined by η (and the corresponding *p*O_2,eff_ via the Nernst equation, Equation (4)) and *p*O_2,ref_, respectively. The equivalence of this can be easily understood by looking at the schematics in Figure 1c, where the ECR phase (II) directly results from the polarization phase (iv). As a jump in *p*O_2_ is omitted, this approach is free of any flush time limitation and enables operating with reduced gas flow rates or even under static conditions, decreasing surface cooling effects and providing strongly enriched flexibility for the study of different atmospheric compositions and *p*O_2,eff_ step sizes. Note that, although the MIEC WE is effectively reduced upon application of a cathodic bias, oxygen is incorporated at the WE surface (oxidation reaction). In the subsequent relaxation step, the reduced MIEC is re‐oxidized. Thus, the net oxygen flux across the WE surface is in the same direction for both, step 1 and 2, as illustrated in Figure 1d (see black arrows). We therefore use the terms oxidation and reduction to refer to the WE surface processes during and following cathodic (η < 0) and anodic (η > 0) polarizations, respectively.

Altogether, BCR delivers several important kinetic parameters (chemical, electrical, and electrochemical surface exchange coefficients, as well as initial and net reaction rates, see below) of one and the same surface (including their activation energies and reaction orders) and a complete picture of the ionic and electronic defect state of the investigated MIEC material system (Δδ, σ) within a single experiment. At the same time, inaccuracies due to finite flush times or not well‐defined starting points of the conductivity relaxation process are avoided, and the influence of voltage‐induced modulations of the surface potential, Δχ(η), on the reaction kinetics can be decoded in a unique manner by comparing kinetic parameters of electrochemical and chemical origin from complementary polarization and relaxation measurements. Furthermore, thorough BCR data analysis allows to separate kinetic contributions of the remaining cell components (CE + electrolyte) and to quantify the voltage‐overpotential conversion efficiency, covering aspects of EIS. All this is demonstrated in the following section. An overview of the accessible parameters and a qualitative benchmark comparison for BCR, ECR, EIS, and polarization measurements is given in the Table S1.

Moreover, BCR captivates by its very simple setup, which consists only of a temperature system (with five electronic contacts), a standard source meter, and a digital relay. This will benefit its fast dissemination and implementation as a new, fast, and cost‐effective standard tool, which can be readily coupled to other in situ techniques such as optical absorption, NAP‐XPS, X‐ray diffraction, spectroscopic ellipsometry, and Raman spectroscopy to provide additional insights on specific reaction intermediates, the structural and surface chemical evolution, etc. Brief amendatory notes on sample and setup requirements for BCR measurements can be found in the Note S1, with different electrode geometries given in Figure S1.

## 3 Results and Discussion

We have studied dense, 120 nm thick sub‐stoichiometric (La,Sr)FeO_3‐δ_ thin films deposited by pulsed laser deposition (PLD) on 5 × 5 mm ionic conducting La_0.95_Sr_0.05_Ga_0.95_Mg_0.05_O_3‐δ_ (LSGM) single crystal substrates (100). SEM images of the top surface and XRD pattern of a fully reduced and fully oxidized sample can be found in Figure S2, revealing small grain sizes, with an average equivalent diameter of about 80 nm, and a (h00) pseudo‐cubic structure [46]. Four 100 nm thick Au top electrodes have been fabricated using photolithography and metal e‐beam evaporation in cleanroom facilities. A porous, paint‐brushed Ag layer served as the counter electrode. We verified that WE surface reactions are limiting the overall oxygen pathway and thus η ≈ *U*_appl_ using electrochemical impedance spectroscopy (EIS) and by comparing conductivity values and the amount of incorporated charge for different effective and reference oxygen partial pressures, see the Note S3 and Figure S3a,c,d). Additionally, we performed gas sensor type measurements, where WE and CE are electrically short‐circuited using an ammeter and the out‐of‐plane current is measured upon performing a switch in the gas atmosphere, as shown in Figure S3b for a jump toward lower *p*O_2_. The negative sign of the current implies that oxygen ions are transported from the WE to the CE through the electrolyte. Thus, the oxygen stoichiometry of the MIEC equilibrates to the lower *p*O_2_ predominantly through the CE rather than through its native surface, as shown by the analysis of the transported charge in the Supporting Information. The fact that the oxygen evolution reaction is “outsourced” to the CE surface confirms that surface reactions of the WE are limiting the overall equilibration process, which is a precondition for the following BCR measurements, performed on LSF thin films.

### 3.1 “1 + 1 = 2”: Added up Information Depth Using BCR

**FIGURE 2:**
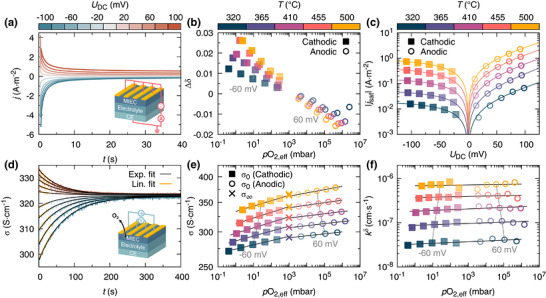
Bias‐triggered conductivity relaxation (BCR) measurements: (a) polarization curves (step 1) and (d) subsequent conductivity relaxation processes (step 2, coloring according to step 1) at 410°C in 1 bar of flowing oxygen for various positive and negative biases. (b) Change in oxygen stoichiometry as a function of effective oxygen pressure and (c) saturation current density as a function of applied voltage, obtained from polarization curves at different temperatures. Log‐log plots of the (e) initial and final conductivity, σ_0_, and σ_∞_, respectively, and (f) the chemical surface exchange coefficient as a function of the initial effective *p*O_2_ inside the MIEC for various temperatures (*p*O_2,final_ =  1000 mbar). Dashed lines in (e,f) are guides to the eye. Values in (e,f) were extracted from relaxation data shown in (d) using exponential fitting curves (black dashed lines). The magnitude of the (preceding) bias is indicated via the symbol's opacity in (b,c,e,f).

The integration of different techniques into a single BCR experiment allows to simultaneously determine all relevant material descriptors for MIET reactions. The rich and abundant information density provided by BCR measurements is summarized in Figure 2, including thermodynamic and kinetic parameters obtained from polarization (top row) and subsequent relaxation (bottom row) measurements.

In a first step, the application of a bias, *U*_DC_, triggers a current of the same polarity, which rapidly decays, as shown in Figure 2a for biases between −100 and +100 mV at 410°C. Additional raw data for other temperatures can be found in Figure S4. Commonly, two figures of interest are extracted from such plots, namely the charge incorporated into the WE, corresponding to the change in oxygen stoichiometry, Δδ, and the saturation current density, *j*_sat_, which is a measure of the net reaction rate. Additionally, we identified that the onset current density, *j*(*t*  =  0) contains entangled information about the kinetic activity of the remaining components of the electrochemical cell, including the electrolyte and the CE, and as such, is a function of the voltage step. This relation can be used to analyze the process of building up the overpotential inside the WE and quantify the voltage‐overpotential conversion efficiency (here ≈95 %), as well as to estimate the error in the incorporated charge, due to a non‐constant leakage current, see Note S5 and the corresponding Figure S6. A more detailed analysis will be the subject of a forthcoming paper.

The relative change in oxygen stoichiometry, Δδ=δ(η≠0)−δ(η=0), obtained via Equation (7), is plotted as a function of the effective *p*O_2,eff_ Equation (4), in Figure 2b. The oxygen off‐stoichiometry varies within a narrow window of −0.02 to 0.03, clearly showing that the overpotential is compensated ionically. In accordance with bulk behavior [47], the oxygen content increases with *p*O_2,eff_ (i.e. Δδ decreases), and the slope steepens with increasing temperature.

The current density‐voltage characteristics are plotted in Figure 2c. An analytical expression for such curves was derived previously for certain limiting situations [48], which is formally equivalent to an exponential Butler‐Volmer type equation:

(10) $$j=j_{0}\;\left[e^{\frac{\alpha_{a}F\left(\eta -U_{0}\right)}{RT}}-e^{\frac{-\alpha_{c}F\left(\eta -U_{0}\right)}{RT}}\right]$$

with the exchange current density, *j*_0_, and a voltage offset, *U*_0_, which here likely corresponds to a thermovoltage due to asymmetric heating in the deployed temperature cell (≲ 10 mV), which renders a general issue for polarization measurements using button heaters. The small thermoelectric voltage adds up to the applied bias and therefore results in an additional slight modification of the steady‐state defect concentrations during the polarization step. However, this contribution, as well as contributions to *j*_sat_ are expected to be negligible for the studied voltage ranges (see Note S4). The coefficients α_*a*_ and α_c_ for the cathodic and anodic branches are functions of the reaction orders of the defects participating in the forward and backward reactions of the ORR, respectively [48]. Good modeling can be achieved for all temperatures using least square fitting, as shown by dashed lines. The corresponding fit parameters are reported in Figure S7a,b. The exchange current density, *j*_0_, rapidly increases with temperature. *j*_0_ is directly related to the electrical surface exchange coefficient, *k* ^q^ = *j*_0_ (2*ec*_O_)^−1^, defined for close to equilibrium conditions (i.e. only small excitation voltages) and contains the total oxygen ion concentration, *c*_O_ [49]. Its temperature evolution is depicted in Figure S7c. As shown in Figure S7a, α_*a*_ ≈ 1 and α_c_ ≈ 0 remain approximately constant with temperature, suggesting the predominance of a single RDS over the analyzed temperature range. In contrast to simple cases in aqueous systems, however, α_c_ ≈ 0 does not imply a diffusion‐limited process but can be the result of two scenarios: either defects participating prior to or during the RDS of the ORR are independent of the overpotential, or more likely, their individual contributions counterbalance each other.

In a second step, the applied overpotential is cut and the effective *p*O_2_ state of the WE re‐equilibrates with the surrounding atmosphere exclusively through its own surface. This relaxation can be followed electrically by rapid digital switching from the out‐of‐plane polarization to the in‐plane resistivity electrode configuration, as sketched in the inset of Figure 2d. Conductivity relaxation curves from various preceding anodic/cathodic biases at 410°C are presented in Figure 2d, for other temperatures, see Figure S4. Examples for normalized conductivity curves are given in Figure S5a,b. The equivalence of BCR and true ECR relaxation transients, based on changes in effective *p*O_2_ vs. atmospheric *p*O_2_, is verified in Figure S8.

As predicted above, the relaxation through the native WE surface is one to two orders of magnitude slower than the outsourced oxygen *charging* of the WE via the CE during the preceding polarization step. Transitions from higher to lower oxygen stoichiometries are accompanied by a decrease in conductivity (reduction, red curves) and vice versa (oxidation, blue curves), as expected for a predominantly *p‐*type conductor. All conductivity relaxation curves within the analyzed temperature range can be well modeled using the exponential Equation (9) (dashed black lines in Figure 2d), providing access to the saturation time, τ, as well as the initial and final conductivity values, σ_0_ and σ_∞_. It is noteworthy that good fitting with a single saturation time is expected exclusively for purely surface‐limited processes [39, 50], which, in combination with shorter polarization than relaxation times, is an additional confirmation that a WE surface reaction limits the cell under the here investigated conditions.

Saturation of all curves at the same σ_∞_ value (± 0.5 S∙cm^−1^) is a good indicator that there are no irreversible changes affecting the materials conductivity during the BCR measurements. Given the very fast electronic switching (≪ 1 s) between out‐of‐plane polarization and in‐plane electrical measurements, σ_0_ approximately equals the equilibrium conductivity for the effective oxygen pressure inside the MIEC during the preceding polarization step. This is confirmed as well by the overlap of conductivity data obtained at 5 and 1000 mbar in Figure S3c for various *p*O_2,eff_ states. The *p*O_2,eff_ and temperature dependence of the electrical conductivity are shown in Figure 2e and Figure S9b, respectively. The measured σ values compare well with literature for highly oxidized LSF [42, 51, 52, 53]. The very low reaction order, $m\;\left(\sigma \right)=\frac{\partial \sigma }{\partial pO_{2}}\;$, of around 0.01 for the cathodic branch, which levels off even further to 0.005 for anodic polarisations (see Figure S10a), is an indicator that the structure is approaching the stoichiometric endpoint with δ  =  0 at 1 bar, where the electron hole concentration is constant and given by the cationic substitution concentration. Going to more reducing biases (thus lower *p*O_2,eff_) and/or lower atmospheric *p*O_2_, the reaction order increases to about 0.15 (see Figure S3c), becoming closer to reported LSF thin film data [54]. For bulk LSF under oxidizing conditions, the pressure dependency is typically in the range of 0.2–0.25 above 600°C [52, 55, 56].

The chemical surface exchange coefficient is obtained via the time constant, *k* ^δ^ = *d*_film_ τ^−1^. Its thermally activated nature is readily visible in Figure 2f, with a strong increase in magnitude going from 320°C to 500°C. In contrast to *j*_sat_, *k* ^δ^ is barely affected by the preceding bias with no significant asymmetry between anodic and cathodic branches, i.e. *k* ^δ^ is independent of the *p*O_2_ step size and its direction (within the studied range), and thus the initial defect state of the bulk, including oxygen vacancy and electron hole concentrations. This means that the duration of the relaxation process is independent of the total amount of oxygen to be incorporated, which confirms the validity of a first‐order kinetic regime Equation (8), i.e., the reaction rate is proportional to the change in oxygen concentration, Δ*c*  = *c*_∞_  − *c*(*t*). Reducing *p*O_2,ref_ on the other hand, leads to a significantly lower *k* ^δ^, as shown in Figure S11e, with a *p*O_2_ reaction order, $k^{\delta }\propto pO_{2}^{m}$, of about *m* ≈ 0.3 (see Figure S11f). This highlights the strong influence of the *p*O_2,ref_ dependent surface state and adsorbate coverage on *k* ^δ^. Similar results have been discussed previously in the literature, including matching *k* ^δ^ values for oxidation and reduction steps in La_0.6_Sr_0.4_FeO_3‐δ_ thin films [54], as well as *k* ^δ^ being independent of the step size Δ*p*O_2_ (while maintaining the final *p*O_2_ constant) in La_0.5_Sr_0.5_CoO_3‐δ_ bulk samples [57, 58].

The ratio of *k* ^δ^ and *k* ^q^ defines the so‐called thermodynamic factor, *w*_O_, a measure of a material's ability to change its oxygen stoichiometry upon changes of the chemical potential (e.g. via the *p*O_2_). By comparing *k* ^δ^ and *k* ^q^, we obtain a value of about 100, which matches well the typical figure for oxides with large chemical capacitance [49]. As an additional kinetic parameter, the initial rate of change, ℜ_ini_, can be obtained from the slope of the relaxation transients close to *t*  =  0 (cf. linear fits with the slope *a*, given by solid orange lines in Figure 2d) [32]. Alternatively, ℜ_ini_ can be estimated from the first term of the series expansion of the derivative of the exponential fitting curve:

(11) $$R_{\mathrm{ini}}\propto a\approx \Delta \sigma \tau^{-1}$$

with Δσ  = σ_∞_  − σ_0_, as confirmed in Figure S9a. Before discussing ℜ_ini_ further in the next section, we analyze the temperature evolution of derived kinetic parameters.

**FIGURE 3:**
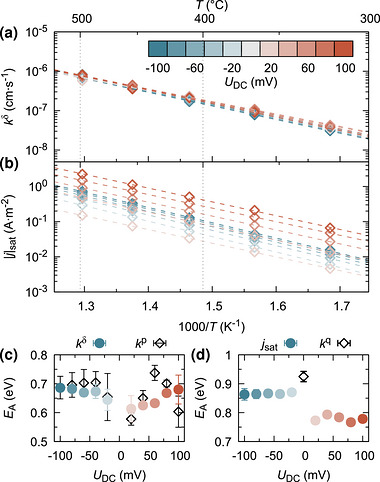
Arrhenius plots for (a) *k* ^**δ**^ and (b) |*j*_**sat**_| for different polarizations at 1 bar of O_2_. The corresponding activation energies are shown in (c, d), respectively. Panel (c) also displays the activation energy for the electrochemical surface exchange coefficient, *k* ^**p**^ (open diamonds), introduced and discussed in section 3.2.

Arrhenius plots for *k* ^δ^ and |*j*_sat_| are presented in Figure 3a,b, respectively. As noted above, *k* ^δ^ values for oxidation (blue) and reduction steps (red) are basically identical and fall onto the same line, with matching activation energies of around 0.65 ± 0.05 eV, as shown in Figure 3c. This value is within the wide range of reported activation energies for LSF, ranging from approximately 0.5 to 1.5 eV for different LSF compositions, and sample types [40, 59, 60, 61]. For |*j*_sat_| we find distinct activation energies for cathodic (blue) and anodic (red) polarizations, see Figure 3d, both slightly higher than the activation energy for *k* ^δ^ and closer to the activation energy of the electrical surface exchange coefficient, *k* ^q^. The pre‐exponential factors from the corresponding Arrhenius laws for |*j*_sat_| and *k* ^δ^ are given in Figure S7d, which in contrast to *E*_A_ present a clear dependence on polarity and magnitude of the bias.

### 3.2 “1 + 1 = 3”: Synergetic Aspects of BCR

In the previous section, we demonstrated the ability of BCR to perform synchronous multi‐parameter characterization within a single measurement. However, the simultaneous determination of multiple material properties enables unique correlations and new insight and thereby exceeds the information depth of the individual approaches. These synergetic aspects of BCR are highlighted in the following.

**FIGURE 4:**
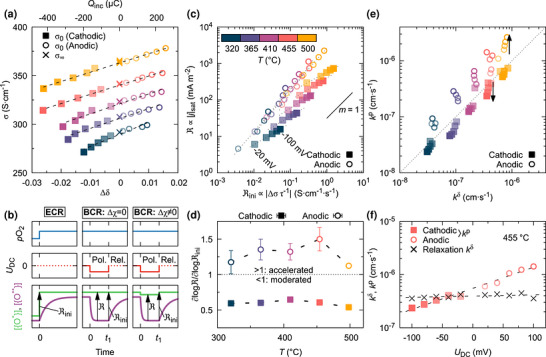
(a) Electronic conductivity of LSF as a function of changes in oxygen off‐stoichiometry. Dashed lines are a guide to the eye. (b) Schematic time profiles of *p*O_2_, *U*_DC_, and defect concentrations of the oxygen intermediate states before and after the RDS, {O*}, and {O**}, respectively, for ECR and *j* − η measurements. If {O*} carries an electronic charge, its concentration is modified upon application of a bias, and Δχ≠0. A dotted line for the voltage indicates open circuit conditions across the device. (c) Correlation between independent measures of the (initial) reaction rate obtained by *j* − η (*y*‐axis) and ECR (*x*‐axis) measurements. Dotted lines are linear fits for cathodic and anodic branches at each temperature, with their slopes being shown in (d). (e) Direct numerical comparison of the electrochemical and chemical surface exchange coefficients, *k* ^p^, and *k* ^δ^, respectively, obtained from *j* − η/titration measurements and the subsequent relaxation processes using the BCR methodology. (f) Bias dependence of *k* ^p^ and *k* ^δ^ at 455°C. The opacity of the symbols indicates the magnitude of the bias voltage in (a,c,e,f).

For instance, by combining titration with conductivity measurements, BCR readily provides the dependence of electronic properties on relative changes in oxygen deficiency, not accessible via standard σ vs. *p*O_2_ experiments (Brouwer analysis). Knowledge of such experimental σ − Δδ relationships, however, is essential for understanding the physical origin of changes in electronic conductivity as well as for the correct mathematical modeling of ECR transients (e.g. lack of this knowledge is one factor limiting ECR measurements to small *p*O_2_ steps, where a linear σ − Δδ relation can be assumed [50]). At high temperatures, we find a linear σ − Δδ dependence, as shown in Figure 4a. This suggests that variations in σ are dominated by changes in the charge carrier density via the oxygen reduction reaction and that the hole mobility is constant and proportional to the slope. The emerging, weak deviation from linearity at lower temperatures likely results from changes in the charge carrier mobility, as reported previously for LSF [62, 63].

In addition to the standard kinetic analysis performed above, BCR enables the direct comparison of kinetic processes triggered by either purely chemical or electrical driving forces. This allows us to unravel the intrinsic effects of an overpotential on the reaction kinetics under current. To the best of our knowledge, a similar approach has not yet been reported in the literature.

We start by looking at the reaction rates ℜ and ℜ_ini_ obtained from polarization and subsequent relaxation curves, respectively. By design of the experiment, the step in µ_O_ between the atmosphere and the MIEC bulk is identical for both reaction rates (cf. Figure 1). Consequently, any difference between ℜ and ℜ_ini_ must be directly linked to the applied voltage and the presence of electrical currents during the first step, due to bias‐induced energy shifts of the work function, variations of the surface coverage of charged adsorbate species (e.g.$\left[O_{\mathrm{ad}}^{2-}\right]$) and resulting changes of the electrostatic dipole layer.) and resulting changes of the electrostatic dipole layer. These effects ultimately alter the reaction intermediate concentrations, as well as the surface electrostatic potential step, and thus the reaction rate.

Let us clear this point by re‐analyzing a traditional ECR step, as well as BCR measurements with and without modifications of the electrostatic surface potential. The corresponding *p*O_2_ and *U*_DC_ profiles (representing the driving forces) are illustrated in Figure 4b. The exact state of the oxygen intermediates participating in the RDS is unknown, and we therefore use {O*} and {O**} to refer to the reactant and product oxygen species of the RDS, respectively, as introduced in [34]. The evolution of their defect concentrations is depicted in the bottom schematics of Figure 4b.

For an ideal ECR experiment, [{O*}] jumps instantaneously to the new equilibrium concentration, while at *t*  =  0, [{O**}] is still in the pre‐equilibrium state and starts transitioning to the new equilibrium with ℜ_ini_. For a BCR experiment with Δχ (η) =  0, the applied overpotential only modifies [{O**}], resulting in a net reaction rate ℜ. The steady state during polarization and the starting point (*t*  =  0) of the subsequent relaxation have the same oxygen intermediate concentrations [{O*}] and [{O**}] and thus ℜ and ℜ_ini_ are equal, as indicated by arrows in the bottom centre schematic of Figure 4b.

For sufficiently large deviations from equilibrium, i.e., large effective *p*O_2_ steps, the net reaction rate can be approximated by the forward reaction rate, $ℜ\approx \vec{ℜ}$ [32]. In such a case, Δχ (η) =  0 is only expected, if no net electric charge is involved prior or during the RDS (that is {O*} is neutrally charged), and changes of the work function are negligible. However, if {O*} is a charged state, e.g., adsorbed superoxide ($O_{2,\mathrm{ad}}^{-}$) is involved as a reactant in the RDS, an electric field and resulting currents can act on {O*}, capacitively modifying its concentration and impeding or enhancing any additional charge transfer across the surface dipole layer during the RDS in a non‐trivial manner. This will affect the reaction rate, ℜ, as schematically drawn in Figure 4b (bottom right). Based on the RDS assumption, changes in {O*} and surface electrostatic relaxation processes take effect quasi‐instantaneously after cutting the applied voltage and {O*} relaxes immediately to the unbiased equilibrium with Δχ(η  =  0) ≡ 0. Thus, opposite to ℜ in the steady state, the initial reaction rate, ℜ_ini_, of the relaxation transient remains unaffected by electrostatic surface effects of the preceding bias. Therefore, true equivalence between the BCR relaxation step and a commensurate ECR pressure step is given as long as a single RDS prevails, as verified in Figure S8.

The experimentally obtained reaction rates ℜ and ℜ_ini_ for LSF are compared in Figure 4c. We find a correlation between these quantities indeed, with distinct power law dependencies $ℜ\propto {ℜ}_{\mathrm{ini}}^{m}$ for the cathodic (oxidation), and anodic (reduction) branches, with *m* < 1 for cathodic and *m* > 1 for anodic biases at all temperatures, see Figure 4d. Upward and downward deviation from *m*  =  1 indicate that ℜ is accelerated upon positive voltages and moderated upon negative ones with respect to ℜ_ini_, revealing a direct potential‐induced electrostatic modulation of the reaction kinetics, in agreement with a recent mechanistic study on LSF, which found hints toward a dependence of the surface potential on the applied bias [64]. We hypothesize that the exponent *m* contains key information about the origin of this effect (e.g. an increase/decrease in surface coverage of negatively charged oxygen adsorbates), but further research is required to elucidate this point and establish a solid, quantitative interpretation of the physical meaning of *m*, e.g. by combining BCR with near ambient pressure XPS (NAP‐XPS) studies [65, 66]. Complementary, the close relation found between ℜ∝*j*_sat_ and ℜ_ini_∝Δστ^−1^ demonstrates that the two individual approaches for the mechanistic interpretation of the ORR, separately developed by Merkle [32] and Schmid [33], are actually two sides of the same coin.

The driving force for net oxygen flux across the WE surface is the difference of the oxygen chemical potential of the WE and the atmosphere, Δ µ_O_ = µ_WE_  − µ_atmo_. Within a first‐order kinetic regime, the flux is directly proportional to Δµ∝Δ*c*  = *c*_∞_  − *c*(*t*). In classic chemical (relaxation) experiments, Δ*c* arises due to a change in the *p*O_2_ (and thus *c*_∞_), while for polarization measurements the atmosphere is maintained (*c*_∞_ =  const.) but the oxygen concentration of the WE, *c*(*t*), is altered (given a surface‐limited overall reaction). Analog to the definition of the chemical surface exchange coefficient, *k* ^δ^, in Equation (8), we can therefore express an electrochemical surface exchange coefficient, *k* ^p^, for polarization experiments. With the flux given by *J*  =  ℜ  = (2*e*)^−1^ *j*_sat_ and the affinity, here Δ*c*, taken from titration measurements via Δ*c*  =  Δδ*V*_uc_^−1^, we can write:

(12) $$k^{p}=J\cdot \Delta c^{-1}=\frac{j_{\mathrm{sat}}d_{\mathrm{film}}}{\int \left(j\left(t\right)-j_{\mathrm{sat}}\right)\mathrm{dt}}\;$$

While *k* ^δ^ describes the kinetic processes originating in a purely chemical gradient across the surface, *k* ^p^ is a descriptor for the modified reaction kinetics under the same chemical gradient but with an additional external electrostatic component, due to the interaction of the applied bias with the intrinsic electrostatic surface potential, as sketched by the two arrows in the bottom right schematic of Figure 4b. As such, *k* ^p^ is the determining kinetic surface parameter for any electrochemical application. Based on the two‐step procedure, BCR provides both the chemical and electrochemical surface exchange coefficients, enabling direct comparison, as shown in Figure 4e. Notably, for small absolute voltages (symbols with low opacity), we find almost matching *k* values, i.e. points falling onto the diagonal. Increasing the bias magnitude affects mainly *k* ^p^, (i.e. vertical changes, as marked with black arrows). This dependence becomes readily visible when plotting *k* ^δ^ and *k* ^p^ against the applied voltage, as depicted in Figure 4f for 455°C and in Figure S12 for all analyzed temperatures. While *k* ^δ^ is approximately constant, *k* ^p^ grows exponentially with *U*_DC_, being smaller than *k* ^δ^ under cathodic polarizations and significantly accelerated under anodic ones. Note that the crossing of the trend lines is slightly offset to below 0 V, which is expected to be an artefact due to setup‐related, small thermovoltages. As for the reaction rate above, we speculate that the drastic influence of the polarity on *k* ^p^ is linked to electrostatic effects, such as coverage changes of charged adsorbates and modifications of the surface dipole layer with Δχ(η)≠0. Thus, these measurements provide direct evidence that a charge transfer reaction must take place before or during the RDS, as otherwise Δχ(η) should vanish (for the forward rate of the RDS) and *k* ^δ^ = *k* ^p^ , independent of η (within a limited η range, see next section).

The temperature dependence of *k* ^p^, accessible by conventional *j* − η/titration measurements, is shown in Figure S13, together with the *k* ^δ^ values from relaxation. The corresponding activation energies, *E*_A_, for different biases are added to Figure 3c (black diamonds). Particularly for oxidation steps (cathodic biases), activation energies obtained from relaxation transients (*k* ^δ^), and titration experiments (*k* ^p^) match very well, pointing toward the same RDS. This demonstrates that electrochemical surface exchange coefficients and corresponding *E*_A_ values can be retrieved from standard *j* − η measurements.

Commonly, the chemical and electrical surface exchange coefficients, *k* ^δ^ and *k* ^q^, are investigated, which correspond to oxygen transport processes arising from purely chemical gradients or small excitation voltages near equilibrium, respectively, and are linked via the thermodynamic factor, i.e. *k* ^δ^ = w_*O*_ *k^q^* (as long as the same basic RDS prevails) [67]. However, for any application operating in the presence of chemical and electrical gradients, such as gas sensing and energy conversion, *k* ^p^, derived from steady‐state polarization measurements, is the relevant kinetic surface parameter. Thus, only *k* ^p^ is a reliable performance indicator for such operation conditions and should be preferred over the two conventional parameters, *k* ^δ^ and *k* ^q^.

Up to this point, we can derive some general conclusions. The combination of electrical and chemical driving forces proved a powerful yet simple approach to study all relevant materials descriptors for MIET reactions. BCR provides access to the bulk oxygen stoichiometry and electrical conductivities, as well as surface kinetic properties, such as net and initial reaction rates and the chemical and electrical surface exchange coefficients. Additionally, we showed that an ‐ here introduced ‐ electrochemical surface exchange coefficient of the WE and corresponding activation energies can be determined from traditional *j* − η measurements, which also cover aspects of electrochemical impedance spectroscopy by containing kinetic information of the counter electrode (via the onset current density). Moreover, we verified the potential of the BCR technique to investigate electrostatic effects arising under current and to separate its contribution to the total reaction rate, opening up new opportunities for detailed studies of surface reactions, while highlighting that Δχ can play a significant role in *j* − η measurements.

Regarding the investigated LSF|LSGM|Ag system, we can conclude that: (i) a first‐order WE surface reaction is limiting the overall electrochemical cell performance. (ii) Initial electronic and ionic bulk defect states (σ and δ) have only a minor effect on the exchange kinetics, in contrast to *p*O_2,ref_, which strongly alters the activity. (iii) Already rather small positive/negative biases accelerate/decelerate the reaction rates, implying that net electric charge is transferred before or during the RDS. Based on these points, we hypothesize that charged oxygen adsorbates play a direct and dominant role in the RDS of the ORR/OER, while a more detailed mechanistic interpretation will be the subject of future work.

### 3.3 Complementary Discussion of the BCR Technique: Applicability, Limits of the Kinetic Regime, and Experimental Restrictions

While this work was focused on LSF, BCR can be applied to any MIEC material system which is suitable for the conventional electrical conductivity relaxation technique, i.e. exhibits a flexible oxygen stoichiometry, which modulates the dominant electrical conductivity. This includes various systems with different defect chemistries, electronic charge compensation mechanisms, and crystal structures, such as commonly investigated cobalt‐based and manganese‐based single perovskite systems, as well as more complex double and triple perovskites (e.g. the superconducting compound YBa_2_Cu_3_O_7‐δ_ (YBCO)) and perovskite‐related structures, such as Ruddlesden‐Popper phase La_2_NiO_4+ δ_ (LNO). Exemplary BCR verification data for LNO and YBCO thin films, with polarization curves showing the expected current decay and conductivity relaxation transients exhibiting single exponential behavior, is presented in Figure S14a,b, demonstrating its general applicability, while a more detailed discussion on these other materials is beyond the scope of this paper.

Note that in its current development state, the BCR method can be used to study material systems, where kinetic processes are limited by a surface reaction. Thus, film thicknesses are restricted to the so‐called critical thickness, approximately separating the kinetic regimes of surface and diffusion limitation, making it interesting for the fundamental study of chemical processes at the surface level. The effect of potential tuning parameters of surface kinetics, such as grain size, strain, surface contaminations, catalysts, gas constituents, etc. can be readily resolved using BCR, as long as MIEC surface limitation prevails. As discussed in more detail in the Note S1, homogenous MIEC electrode polarization during step one of BCR measurements is crucial. This can be achieved by tuning the metallic top electrode geometry to accommodate different ratios of electronic in‐plane conductivity and out‐of‐plane oxygen activity of the MIEC, as has been previously discussed for electrochemical polarization geometries [68, 69]. For materials with moderate in‐plane electronic conductivity, interdigitated, open grid electrodes may be a suitable solution to establish homogeneous surface equipotential conditions. In this work, the equipotential was qualitatively assessed optically via the uniform color change across the full LSF layer. To mitigate complications arising from a potentially inhomogeneous polarization along the MIEC surface for materials with low electrical conductivity, but at the cost of losing electronic information, we suggest that one may be able to follow the chemical relaxation process via out‐of‐plane open‐circuit voltage measurements (i.e. voltmeter mode) using a single, porous current collector layer across the full top surface, as will be thoroughly tested and evaluated in a future work.

For reliable four‐point measurements, the in‐plane electronic conductivity in the MIEC must exceed sufficiently the ionic conductivity of the electrolyte (and the MIEC itself) to avoid parallel ionic current pathways during the conductivity relaxation measurement, ensuring that the measured change in conductivity is dominated by oxygen stoichiometry modulation of the MIEC.

BCR measurements require sufficiently high out‐of‐plane ionic current through the solid‐state electrolyte (sourced at the counter electrode) to modify the oxygen stoichiometry of the MIEC thin film. However, this current decreases exponentially with temperature, limiting BCR measurements to temperatures approximately above 250°C–300°C, which is equivalent to the temperature limit of the conventional polarization and titration approaches. The current response upon cooling (with an applied voltage of 100 mV) is shown in Figure S15. As ionic diffusion through the electrolyte is becoming a relevant polarization contribution at low temperatures, going to counter‐electrode supported cells with a thin film electrolyte may allow to extend the low temperature characterization window. On the other hand, there is no intrinsic limitation toward the high temperature applicability of the BCR method.

**FIGURE 5:**
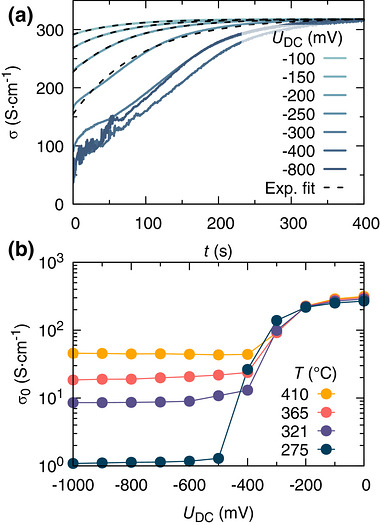
(a) LSF relaxation processes at 410°C for large chemical driving forces: with increasing *U*_**DC**_, transients deviate from the expected behavior for a linear kinetic regime of the oxygen surface exchange, i.e. following a simple exponential curve. (b) Temperature‐dependent saturation of the initial conductivity, σ_0_, at strong cathodic polarizations.

To conclude the introduction of this novel technique, we look at the physicochemical and experimental limitations of bias‐triggered conductivity relaxation. The above analyzed bias range was limited to ± 100 mV, corresponding to changes of more than three orders of magnitude in effective *p*O_2_. All resulting transients obeyed single exponential behavior, a clear sign for a linear reaction order and the surface limited regime. Exceeding this bias range and thus triggering much larger *p*O_2_ steps, we can analyze the validity range for these model assumptions. In Figure 5a we analyze the relaxation from different cathodic polarization steps down to −800 mV. Down to −200 mV, transients can be well described using an exponential function (i.e. Equation (9), see dashed lines in Figure 5a). Notably, an overpotential of −200 mV at this temperature corresponds to a change in *p*O_2_ over six orders of magnitude, i.e., from 10^−3^ to 10^3^ mbar. In contrast to classical ECR experiments, BCR is not affected by any reactor flushtime, and therefore even such large steps can be described within a linear kinetic regime, as indicated by the simple exponential behavior.

Further decreasing the overpotential, deviations become gradually more severe and more complex—a clear sign of a collapse of the first‐order approximation. The origin of the transition from linear to non‐linear kinetics remains unclear, but may include a too large step in Δc, complex interactions between relatively high concentrations of charged surface species (compared to the effective concentrations within the bulk) and the applied voltage, the violation of the single RDS assumption, intensifying contributions of diffusion and other elementary processes, etc.

At −400 mV and below, the initial stage is highly turbulent and conductivity measurements become subject to noise, which was not observed previously. Where fitting was not applicable, the initial conductivity, σ_0_, was determined from the first measured point after switching the electrode configuration. σ_0_ saturates below a temperature‐dependent polarization level, as clearly seen in Figure 5b. Notably, this saturation is independent of the atmospheric pressure, see Figure S3c. While transitions from predominantly hole conduction under oxidizing conditions to electron governed electrical conductivity under highly reducing conditions are commonly found for LSF and other members of the family of oxides, the here observed levelling off is expected to be an experimental artefact: with increasing deviation from equilibrium, reaction rates can alter and other, previously neglectable kinetic contributions may become crucial, such as the reaction kinetics of the counter electrode, as schematically illustrated in Figure S16, while at the same time, the reduced electrical conductivity paired with an accelerated exchange activity exceeding the linear regime, may obstruct homogenous polarization of the MIEC surface for the given electrode geometry. Hence, the efficiency of translating the applied voltage into a uniform overpotential inside the WE is reduced and an increase in the voltage magnitude does not further change the oxygen stoichiometry of the MIEC, resulting in the observed saturation in conductivity. Note that further experimental proof would be needed to precisely understand the saturation of the conductivity and the absence of the p‐to‐n transition in the presented measurements, which is planned for future work using a tuned counter electrode with higher oxygen exchange activity (i.e. increased active area, or faster intrinsic kinetics) and reduced top electrode spacing. Nevertheless, this unexpected behavior occurs for large polarizations well beyond the linear kinetic regime and therefore does not impact the operational window of interest for BCR measurements.

## 4 Conclusions

We have developed bias‐triggered conductivity relaxation (BCR) as a novel tool to study kinetic processes and simultaneously retrieve thermodynamic insight into mixed ionic electronic conducting oxides. The combination of electrochemical titration and in‐plane resistivity measurements and the replacement of steps in atmospheric *p*O_2_ with equivalent electrochemical ones, was demonstrated to provide highly efficient access to all relevant parameters for the analysis of oxygen transport reactions and extend measurement flexibility and precision. We used BCR to perform a standard kinetic analysis of LSF thin films. A comparison of our results with existing literature was conducted to ascertain the validity of the technique. The unprecedented correlation of electrochemical and chemical measurements revealed the direct biased‐induced influence on reaction kinetics. With the introduction of the electrochemical surface exchange coefficient, *k* ^p^, we provide the relevant kinetic parameter for applications operating in the presence of mixed electrical and chemical gradients, such as electrochemical gas sensors, SOFC/SOEC electrodes, and oxygen ion batteries. Moreover, we uncovered that the electrochemical surface exchange coefficient, as well as a kinetic descriptor of the counter electrode, are hidden in conventional current density‐overpotential measurements. We anticipate that the simple implementation and rich information depth of BCR, combined with experimental advantages over conventional techniques, will result in rapid and widespread dissemination and foster the development of materials for sustainable energy applications.

## 5 Experimental Section

La_0.6_Sr_0.4_FeO_3‐δ_ (LSF) thin films of 120 nm thickness were grown at TU Wien by pulsed laser deposition (PLD) on 5 × 5 mm La_0.95_Sr_0.05_Ga_0.95_Mg_0.05_O_3‐δ_ (LSGM) single crystal substrates (100) [70, 71]. The LSGM electrolyte shows predominant ionic conductivity [72]. Metallic Ti(5 nm)/Au (100 nm) top electrodes were fabricated in CNRS' Nanofab clean room facilities using photolithography and evaporation. Gold was selected as the current collector, as it does not catalytically accelerate the ORR on MIEC oxide surfaces. The width and spacing of the metallic stripes is ≈ 0.7 mm. The porous and active Ag counter electrode was fabricated by brush‐paint.

Phase and orientation of LSF thin films were characterized by X‐ray diffraction (XRD) in the θ − 2θ configuration using a Bruker D8 Advance series II diffractometer (CuKα radiation). The surface homogeneity was studied using an FEG ZEISS GeminiSEM 300 microscope in secondary and backscattered electron mode with an accelerating voltage of 3 kV in high vacuum. Image analysis was performed using ImageJ and the Trainable Weka Segmentation plugin.

Functional characterization was performed using current‐overpotential, four‐point electrical resistivity, and electrochemical impedance spectroscopy (EIS) measurements using a Keithley 2400 sourcemeter and a Solartron 1260 impedance analyzer. For in situ measurements, the sample was placed onto a 1/2″ ceramic heating stage of a high‐temperature cell (Nextron), equipped with six electrical probes. A Si substrate with an Ag paint‐brush layer was placed in between the heater and the sample to provide electrical contact to the counter electrode. The temperature at the sample position was calibrated beforehand using a Pt100 thermocouple. However, a certain uncertainty in sample temperature is inherent to such a type of setup, as well as a temperature gradient across the cell. Annealings were performed in a dynamic, dry, mixed O_2_/N_2_ atmosphere at 1 atm and a constant flow rate of 200 mL/min, whereas the oxygen partial pressure was controlled via the flow ratio of pure oxygen to nitrogen gas. The *p*O_2_ was recorded at the exit of the chamber using a Rapidox 2100 gas analyzer. The full setup (including cell temperature, atmosphere, electrical sample configuration via a digital relay, and different measurement types, incl. polarization, conductivity, EIS) was automatically controlled and monitored using a home‐written LabVIEW program.

## Author Contributions

A.St. developed the original concept and methodology, built the experimental setup, performed the measurements, analyzed the data, and wrote the manuscript. A.Sch. and A.St. prepared the samples. A.R. assisted the measurements. A.St, A.Sch., and J.F. critically discussed the results. A.S., A.B., and J.F. acquired the funding for research and personnel. All co‐authors contributed to the revision of the manuscript.

## Additional Information

A pre‐print of this work was published on arXiv available under https://arxiv.org/abs/2601.1580.

## Conflicts of Interest

The authors declare no conflicts of interest.

## Supporting information




**Supporting File**: adma73869‐sup‐0001‐SuppMat.pdf.

## Data Availability

The data that support the findings of this study will be made available in Zenodo under https://doi.org/10.5281/zenodo.18335600.
