# Supplementary material for: Bias‐Triggered Conductivity Relaxation (BCR): A Unique Tool to Simultaneously Investigate Thermodynamics, Kinetics, and Electrostatic Effects of Oxygen Reactions in MIEC Thin Films
Source: Adv Mater. 2026 Jun 30;38(43):e73869. doi: 10.1002/adma.73869 (PMC13432353; doi:10.1002/adma.73869)
Supplement: Supplementary file 1 — Supporting File: adma73869‐sup‐0001‐SuppMat.pdf. [file ADMA-38-e73869-s001.pdf]

# Supporting Information:

# Bias-triggered conductivity relaxation (BCR): a unique tool to simultaneously investigate thermodynamics, kinetics and electrostatic effects of oxygen reactions in MIEC thin films

Alexander Stangl,[1,2,3*] Alexander Schmid,[4] Adeel Riaz,[3] Jürgen Fleig[4] and Arnaud Badel[5]

[*] alexander.stangl@tuwien.ac.at
[1] Atominstitut, TU Wien, 1020 Vienna, Austria
[2] Université Grenoble Alpes, CNRS, Grenoble INP, Institut Néel, 38000 Grenoble, France
[3] Université Grenoble Alpes, CNRS, Grenoble INP, LMGP, 38000 Grenoble, France
[4] Institute of Chemical Technologies and Analytics, TU Wien, 1060 Vienna, Austria
[5] Université Grenoble Alpes, CNRS, Grenoble INP, G2ELab – Institut Néel, 38000 Grenoble, France

## Supporting Information Note 1: Sample and setup requirements for BCR

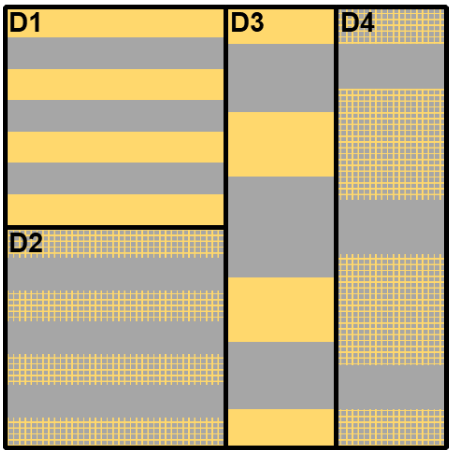


Figure S1: 10×10 mm sample with four different device geometries with open grid (D2, D4) and closed layer (D1, D3) metallic top electrodes for testing purposes. In this work, squared, 5×5 mm devices of type D1 with closed layer electrodes were used.

Obtaining meaning full data via the BCR technique based on the combination of polarization and conductivity measurements requires specific materials properties and precautions in the electrode design.

Homogenous polarization of the working electrode and effective current collection is limited to an area in proximity to the metallic top electrodes. This area depends on the ratio of in-plane electrical conductivity and out-of-plane oxygen activity of the WE, defining a material-specific screening length. On the other hand, four-point electrical resistivity measurements require a certain electrode spacing to provide adequate results. Furthermore, oxygen exchange may be (partially) blocked underneath the metallic current collector. Thus, switching between open grid and closed layer designs and different sample geometries, as shown in Figure S1 allows to vary the accessible WE surface and adapt for its total exchange activity. This gives a parameter to tune the ratio of open surface WE activity and full

area CE activity and ensure WE surface reactions are rate limiting (as long as MIEC ionic bulk diffusivity and electronic conductivity are fast enough to compensate for longer in-plane diffusion lengths).

Sufficiently high in-plane electronic conductivity of the MIEC is not only important for its homogeneous polarization during step 1, but also for precise conductivity measurements during step 2: a high ratio of electronic (MIEC) vs. ionic (MIEC and electrolyte) in-plane conductivity avoids parallel ionic current pathways through the electrolyte (or the MIEC itself), which would interfere with the accurate modelling of the relaxation transients for the determination of kinetic parameters.

Note, that the presence of the current collector may influence the observed kinetics, based on catalytic influences, contaminant interactions, introduction of impurities, space charge modifications and surface blocking effects. However, these effects are expected to be equally relevant during polarization as well as relaxation processes and thus, are not thought to contribute to the observed differences.

Temperature gradients across an electrochemical cell, as for example common for environmental temperature cells with asymmetric heating, can produce thermovoltages in the range of tens of mV. This not only affects small polarization steps, but also leads to unequal equilibrium states for the un-biased but closed-circuit out-of-plane polarization configuration and the in-plane electrical resistivity configuration and has to be considered in the calculation of $p\mathrm{O}_{2,\mathrm{eff}}$.

## Supporting Information Note 2: Comparison of BCR with conventional techniques

BCR holds several key advantages compared to conventional chemical, electrical/electrochemical experiments, with extended information depth, improved time resolution, mitigated limitations, and simple and cost-effective experimental and data analysis.

| | Benchmark | ECR | $j-\eta$ | EIS | BCR |
|---|---|---|---|---|---|
| Kinetics | Chemical surface exchange coefficient, $k^{\delta}$ | best | poor | poor | best |
| | Electrical surface exchange coefficient, $k^{\mathrm{q}}$ | poor | best | best | best |
| | Electrochemical surface exchange coefficient, $k^{\mathrm{p}}$ | poor | best * | poor | best |
| | Net reaction rate, $\Re$ | poor | best | poor | best |
| | Initial net reaction rate, $\Re_{\mathrm{ini}}$ | best | poor | poor | best |
| | Decouple influence of $\chi(\eta)$ | poor | poor | poor | best |
| | Counter electrode kinetics | poor | best * | best | best |
| Thermo-dynamics | Electrical conductivity, $\sigma$ | best | poor | poor | best |
| | Change in oxygen off-stoichiometry, $\Delta\delta$ | poor | best | best | best |
| | Thermodynamic factor, $w_{\mathrm{O}}$ | poor | medium | medium | best |
| Experimental / Analysis / Costs | Flush time limitation | poor | best | best | best |
| | Electrostatic coupling of bias with defects | best | poor | poor | best |
| | Atmosphere flexibility | medium | best | best | best |
| | Time resolution | medium | best | poor | best |
| | Setup / operational costs | medium | best | medium | best |
| | Experimental complexity | best | best | medium | best |
| | Analysis complexity | best | best | medium | best |

Table S1: Qualitative comparison of novel bias-triggered conductivity relaxation (BCR) with established electrical conductivity relaxation (ECR), current-over potential ($j-\eta$, incl. titration) and electrochemical impedance spectroscopy (EIS) in terms of accessible kinetic and thermodynamic parameters and general experimental aspects. Rating is performed using three levels: ■: best/highly suitable; ■: medium/restricted; ■: poor/not applicable. (*) indicates that $j-\eta$ are capable to deliver on these information, which was only established within this work. Note that this comparison is intended to provide a simple graphical overview over the advantages and drawbacks of the individual techniques and ratings are somewhat arbitrary and relative between the compared techniques.

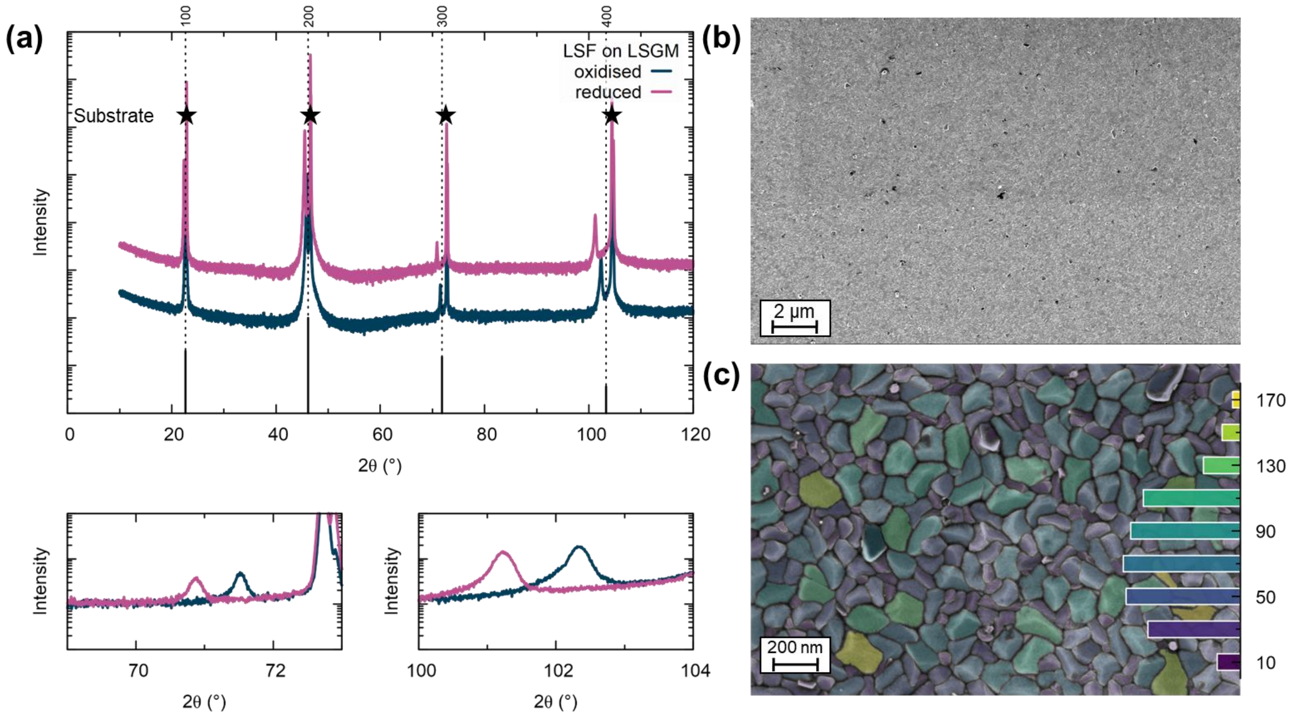


Figure S2: (a) X-ray diffractograms of fully oxidised and reduced (La,Sr)$FeO_{3-\delta}$ thin films grown on (100) $La_{0.95}Sr_{0.05}Ga_{0.95}Mg_{0.05}O_{3-\delta}$ (LSGM) single crystal substrates. The LSF films exhibit a pseudo-cubic structure with (h00) orientation (ICDD: 00-069-0127). The lattice parameter is sensitive to the oxygen stoichiometry, as clearly observable in the magnified regions in the two lower panels for the fully reduced and fully oxidised films, achieved by annealing in low and high $pO_2$, respectively. (b) Low and (c) high magnification scanning electron microscopy images (secondary electron mode) of LSF top surface. The false colour in (c) indicates the grain size, with the corresponding histogram of the distribution of the equivalent diameter (assuming circular grains), with an average $d_{eq} \approx 80$ nm.

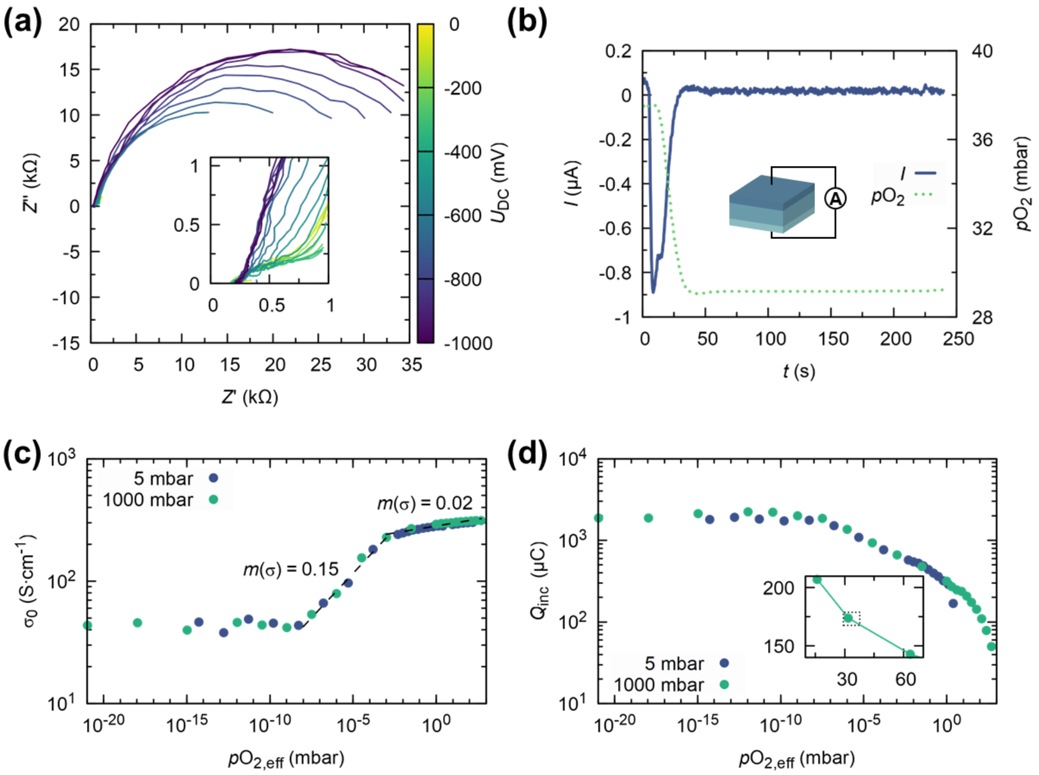


Figure S3: (a) Electrochemical impedance spectroscopy (EIS) 410 °C in 1 bar of $O_2$. (b) A gas-sensor type test, where current is measured across an electrochemical device upon a change in $pO_2$. (c) The initial conductivity, $\sigma_0$, and (d) incorporated charge, $Q_{\text{inc}}$, obtained via bias-triggered conductivity relaxation (BCR) measurements, as function of the effective oxygen partial pressure. Note that $Q_{\text{inc}}$ is not an absolute measure. Therefore, the data set for 5 mbar was shifted upwards to account for the different starting point in oxygen off-stoichiometry.

## Supporting Information Note 3: verification of WE surface limited regime

Figure S3 presents the experimental verification that a working electrode (WE) reaction is overall rate limiting and consequently an applied bias is (almost) fully translated into an electrochemical overpotential using four different approaches. Electrochemical impedance spectroscopy (EIS) data for a LSF thin film, obtained at 410 °C in 1 bar of $O_2$, is shown in Figure S3(a). The very large semi-circle corresponds to the LSF electrode. The inset reveals the very minor electrolyte and counter electrode (CE) polarization contributions of approximately 200 Ω (< 1 %) and 500 Ω (≈ 1-2 %), respectively.

The initial conductivity, $\sigma_0$, and incorporated charge, $Q_{\text{inc}}$, obtained via bias-triggered conductivity relaxation (BCR) measurements are shown in (c) and (d), respectively, as function of the effective oxygen partial pressure. The preservation of the same curvature and overlap of data points measured at different oxygen pressures inside the chamber ($pO_{2,\text{ref}}$), confirm that the applied bias is fully converted into an overpotential, which modifies the oxygen chemical potential of the WE. The levelling off of $\sigma_0$ and $Q_{\text{inc}}$ at low $pO_{2,\text{eff}}$ ($\lesssim 10^{-10}$ mbar) is likely a measurement artefact, as discussed in the main text.

Additionally, we introduce a gas-sensor type test, as shown in Figure S3(b), for the convenient confirmation of WE surface limitation. Here, WE and CE are electrically short-circuited using an ampere meter and the out-of-plane current is measured upon performing a switch in the gas atmosphere. The negative current indicates that the mixed ionic electronic conducting working electrode equilibrates (at least partially) to the new, lower $pO_2$ through the counter electrode, *i.e.* the oxygen evolution reaction is outsourced to the CE. Some oxygen may still be incorporated through the native WE surface.

But that this amount is small can be verified by comparing the area under the $I(t)$ curve with $Q_{\mathrm{inc}} = -12\,\mu\mathrm{C}$ with the dependence of $Q_{\mathrm{inc}}$ on the effective $p\mathrm{O}_2$, as shown in Figure S3(d). The dotted rectangle in the inset of (d) marks the step as performed in (b). By linear interpolation we obtain an expected change of $Q_{\mathrm{inc}} = -11\,\mu\mathrm{C}$ (height of the rectangular). Within the error of this analysis, this figure is ident to the value reported above, confirming that most of the oxygen required for the stoichiometric change of the WE is incorporated through the CE. Note that as the $p\mathrm{O}_2$ sensor is mounted at the gas exit of the sample chamber, a delay in the signal can be observed in (b). On the other hand, the finite transition width, due to the low oxygen gas flow used here (200 ml $\mathrm{min}^{-1}$), would indicate a potential flush time limitation for conventional ECR measurements for samples with fast kinetics.

## **Supporting Information Note 4: thermoelectric voltages**

The use of a button cell with asymmetric heating of the sample, generally results in a temperature difference between the top working electrode (cold side) and the bottom counter electrode (hot side), which is in direct contact to the heater. This temperature difference causes small thermoelectric voltages ($\lesssim$ 10 mV). This voltage becomes relevant once top and bottom electrode are electrically connected during polarization (step 1), where it causes a small additional shift of the steady state defect concentrations of the MIEC. For the relaxation step, and thus $\sigma_0$ and $\mathrm{k}^{\delta}$, it is only relevant in the sense that it had slightly modified the pre-relaxation steady state, while neither the transient itself nor the saturation equilibrium are affected.

The very good overlap of $\sigma_0$ data for different tuples of $(p\mathrm{O}_2, \eta)$, see Figure S3(c), however, indicates that the thermovoltage has negligible influence on the defect concentrations under the studied conditions. Furthermore, as shown in Figure S3(b), the out-of-plane current decays to almost zero under closed circuit conditions. Here, the thermovoltage drives the measured saturation current of ~20 nA, which corresponds to a current density of about $10^{-5}$ $\mathrm{A{\cdot}m^{-2}}$. This value is two to three orders of magnitude smaller than the saturation currents, $j_{\mathrm{sat}}$ under external bias and is therefore not expected to significantly influence the obtained results for the studied voltage ranges. We anticipate that the thermovoltage will become relevant for biases below |20| mV, which requires a setup with symmetric heating.

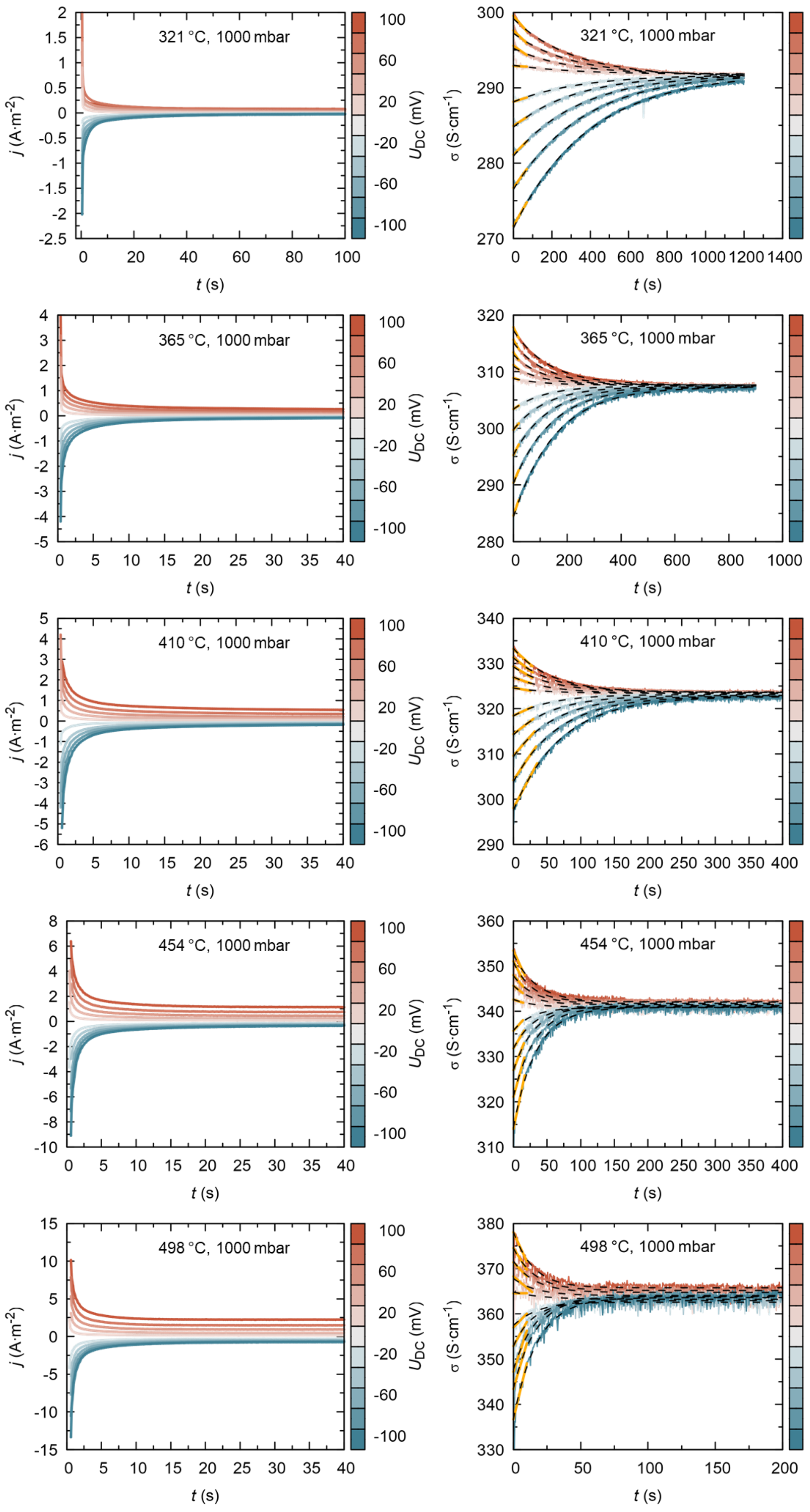


Figure S4: Bias-triggered conductivity (BCR) measurements: polarization (left) and subsequent relaxation (right) curves at different temperatures and various cathodic and anodic biases.

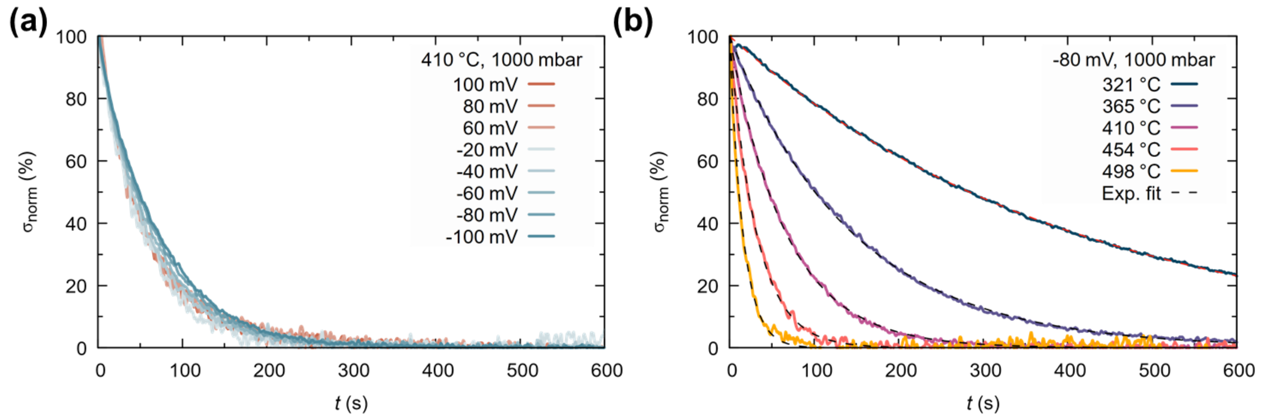


Figure S5: Normalized conductivity relaxation curves: (a) at 410 °C following different bias voltages and (b) at different temperatures after a polarization bias of -80 mV.

## Supporting Information Note 5: Counter electrode + electrolyte kinetics

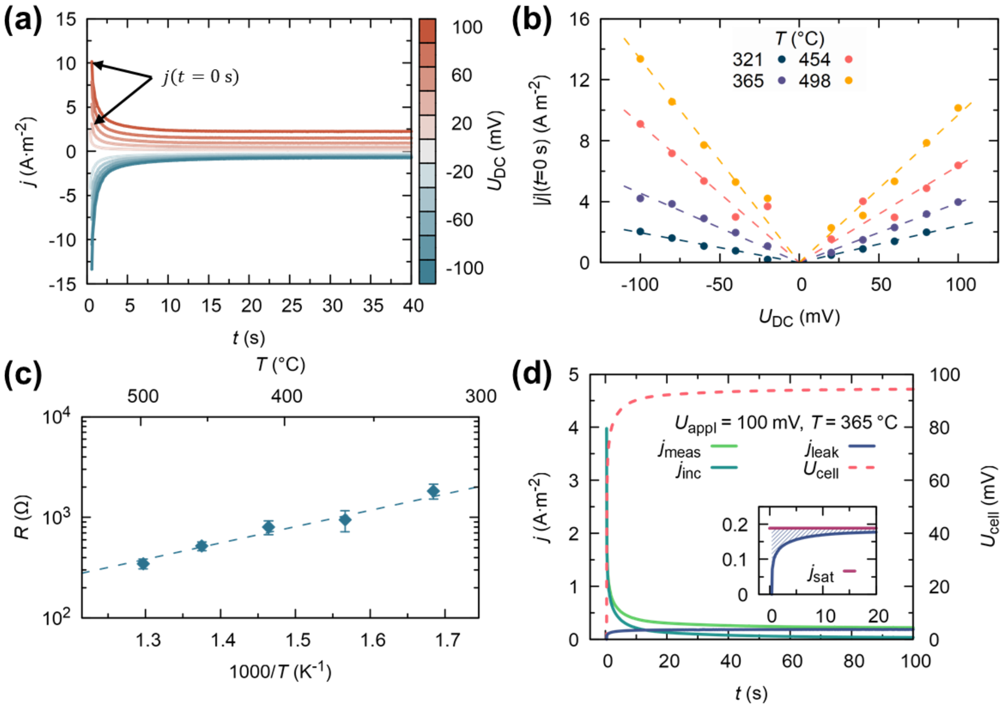


Figure S6: (a) $j(t)$-curves with marked onset current density, $j(t = 0\text{ s})$. (b) Ohmic characteristic of $j(t = 0\text{ s})$ at various temperatures. (c) Arrhenius plot of entangled electrode and electrolyte resistance, obtained from (b). (d) Analysis of time evolution of cell voltage (right *y*-axis) and different current contributions (left *y*-axis). The marked area in the inset highlights the *forgotten* charge, when assuming a constant leakage current for all $t$.

$j(t)$-curves, as shown in Figure S6(a), are commonly evaluated in terms of saturation current densities as well as transported charge (*i.e.* the area between $j_{\text{meas}}(t) - j_{\text{sat}}$). Here we use additionally the onset current density, $j(t = 0\text{ s})$, as a measure of the electrochemical activity of the counter electrode and the electrolyte and further, to evaluate the two different current contributions: namely the one leading to a change in the oxygen stoichiometry ($j_{\text{inc}}(t)$) and the current density corresponding to the leakage flux of oxygen through the WE surface. The $j(t = 0) - U$ plot shown in Figure S6(b) reveals an ohmic characteristic. Thus, the slope is inversely proportional to a resistance. At 450 °C, this resistance matches very well the sum of electrolyte and counter electrode contributions as obtained by EIS, see

Figure S3(a), and therefore supports our interpretation of $j(t = 0\ \mathrm{s})$. The temperature evolution of $R$ is shown in Figure S6(c).

Using the linear $j(t = 0) - U$ dependence (with the slope $m$) from Figure S6(b) and the Butler-Volmer-type dependence of the saturation current density, as presented in the main text, we can approximately analyse the process of building-up the cell voltage, *i.e.* the overpotential in the WE. The overpotential equals the applied voltage minus a voltage drop due to the flow of a current through the counter electrode and the electrolyte, thus we can write: $\eta(t) = U_{\mathrm{cell}}(t) \approx U_{\mathrm{appl}} - U\big(j(t)\big)$, with $U\big(j(t)\big) = j(t)/m$ obtained from the dependence shown in (b). The time evolution of the overpotential (pink dashed line) is shown using the right *y*-axis in Figure S6(d). It sharply rises at $t = 0\ \mathrm{s}$ and saturates at around 95 mV (*i.e.* 95 % of the applied voltage). This simple method allows to quantify the actual overpotential in the presented sample, without the need for additional characterisation steps, such as electrochemical impedance spectroscopy (EIS).

The rising WE overpotential corresponds to a step in oxygen chemical potential across the WE surface, which creates a driving force for net oxygen flux and therefore triggers a leakage current, $j_{\mathrm{leak}}(t)$. The magnitude of the leakage current can be estimated based on the actual cell voltage, $U_{\mathrm{cell}}(t)$, using the Butler-Volmer equation. The time evolution of the leakage current density is shown in (d) and magnified in the inset. Compared to assuming a constant leakage current density (*i.e.* $j_{\mathrm{leak}}(t) = j_{\mathrm{sat}}$), a more precise incorporation current density can be defined via $j_{\mathrm{inc}}(t) = j_{\mathrm{meas}}(t) - j_{\mathrm{leak}}(t)$. Thus, the marked area in the inset of Figure S6(d), between $j_{\mathrm{leak}}$ and $j_{\mathrm{sat}}$, corresponds to additional charge incorporated into the MIEC, which was previously not considered. For the LSF system studied here, the correction was found to be in the range of a few %.

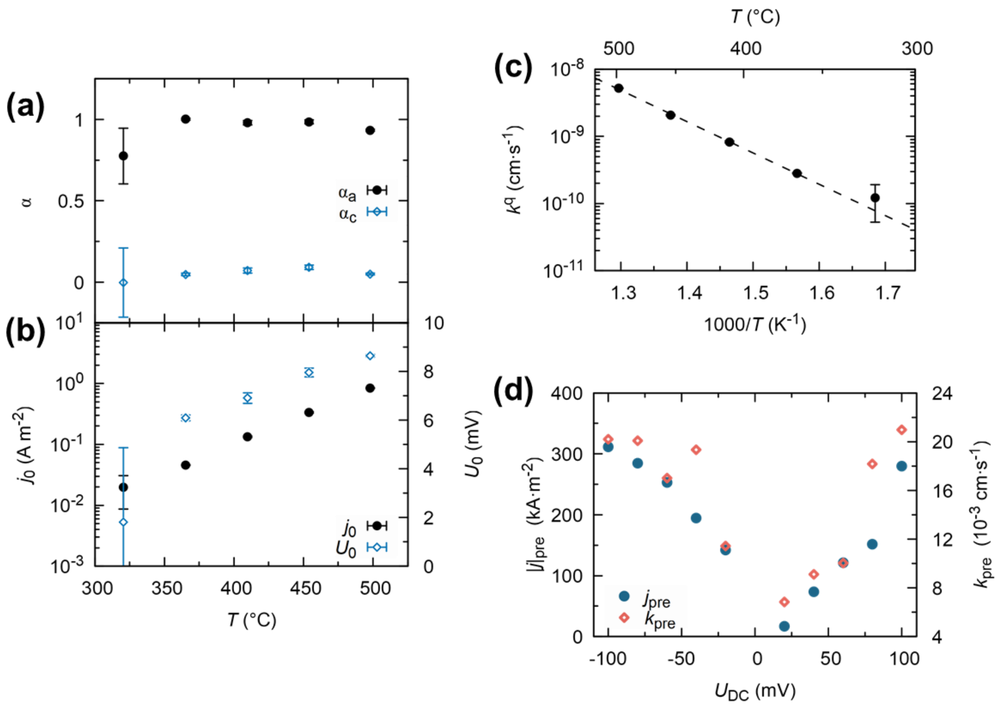


Figure S7: (a & b) Fit parameters for the exponential Butler-Volmer type equation at different temperatures. (c) Arrhenius plot of electrical surface exchange coefficient, $k^{\mathrm{q}}$. (d) Bias dependence of the pre-exponential factors

from Arrhenius-law fittings of the saturation current (left *y*-axis) and the surface exchange coefficient (right *y*-axis).

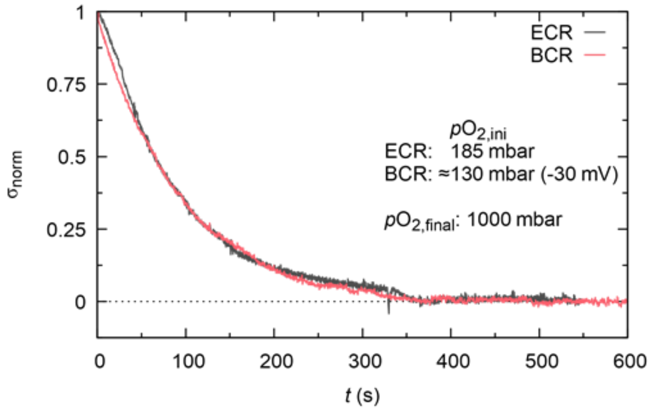


Figure S8: Normalized conductivity relaxation transients obtained from standard ECR measurement via a change in atmospheric $pO_2$ and via novel bias-triggered conductivity relaxation, whereas the initial $pO_{2,\mathrm{eff}}$ inside the MIEC was set via an applied voltage across the electrochemical cell at $t < 0$.

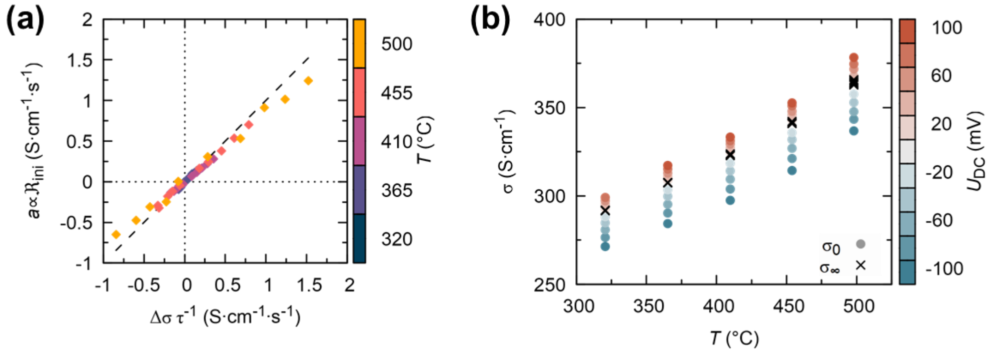


Figure S9: (a) Correlation between the linear slope, $a$, of the initial stage of the relaxation transients and the ratio of the change in electrical conductivity, $\Delta\sigma$, and the exponential saturation time, corresponding to the linear term of the series expansion of the exponential fitting curve. The dashed line marks identity. (b) Temperature dependence of the electrical in-plane conductivity under different polarizations in 1 bar of oxygen.

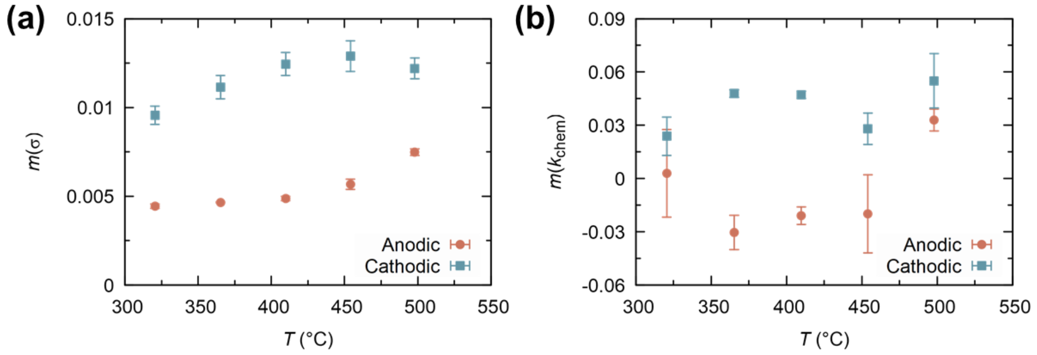


Figure S10: Pressure dependence of (a) the electrical conductivity and (b) the surface exchange coefficient of LSF thin films at different temperatures for anodic and cathodic polarizations.

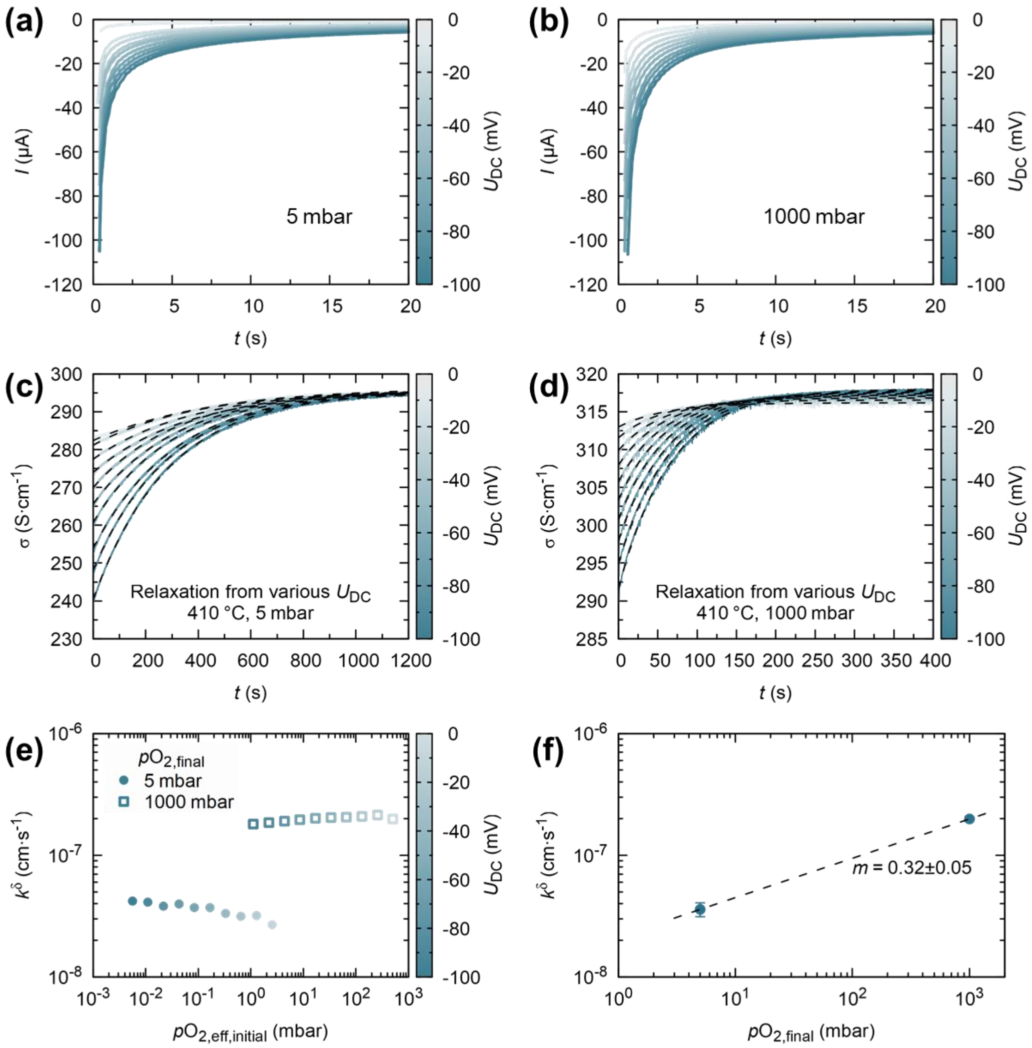


Figure S11: (a & b) Polarization and (c & d) subsequent relaxation steps at 5 and 1000 mbar, respectively at 410 °C. (e) The chemical surface exchange coefficient, extracted from (c & d), is almost invariant to the step size but it is strongly lowered with decreasing $p\mathrm{O}_{2,\mathrm{final}} = p\mathrm{O}_{2,\mathrm{atmosphere}}$ inside the chamber. The $p\mathrm{O}_2$ reaction order of the chemical surface exchange coefficient is approximately 0.3 as shown in (f).

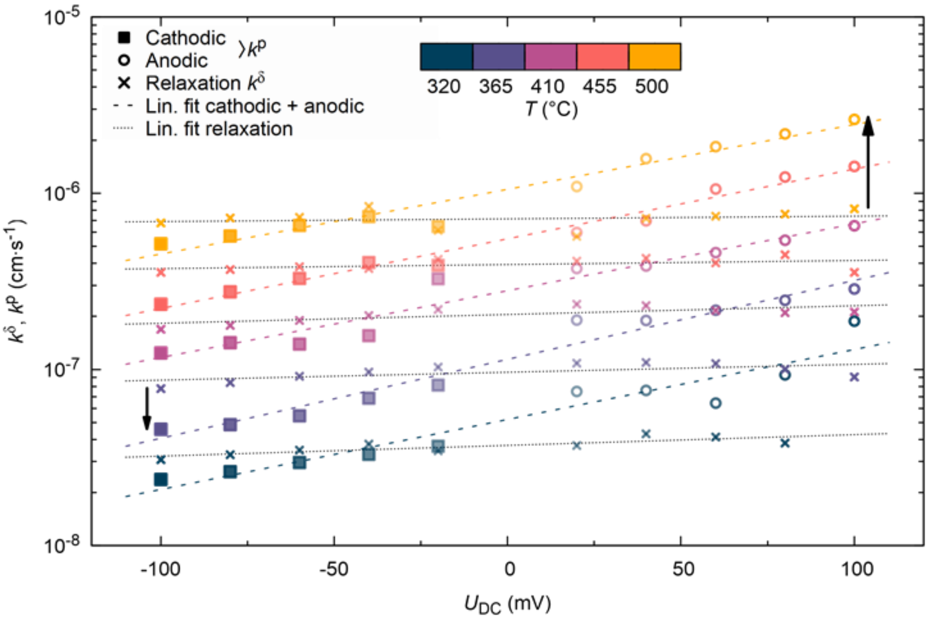


Figure S12: Bias dependence of the electrochemical and chemical surface exchange coefficients, $k^{\mathrm{p}}$ and $k^{\delta}$, obtained from $j-\eta$ measurements and the subsequent relaxation processes. Under cathodic polarizations, $k^{\mathrm{p}}$ is slower compared to $k^{\delta}$, while positive voltages accelerate the surface exchange coefficient compared to the bias-free relaxation process, as marked with black arrows. $k^{\mathrm{p}}$ varies exponentially with voltage (dashed lines), while $k^{\delta}$ is independent of the preceding voltage (dotted lines). The crossing of the two linear trends are observed for all temperatures below 0 V, which is likely linked to thermovoltages due to the deployed temperature cell and asymmetric heating.

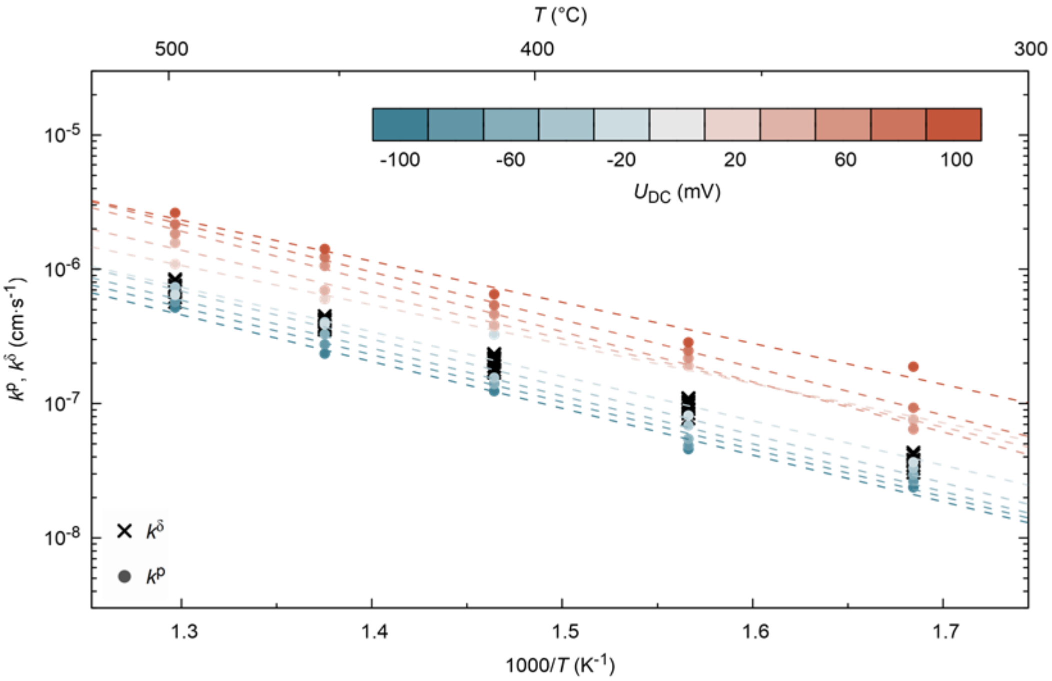


Figure S13: Arrhenius plot of the electrochemical surface exchange coefficient, $k^{\mathrm{p}}$, obtained via titration and $j-\eta$ measurements for different polarizations, compared to the chemical surface exchange coefficient, $k^{\delta}$ from relaxation processes. The activation energies are compared in the main text as function of the polarization.

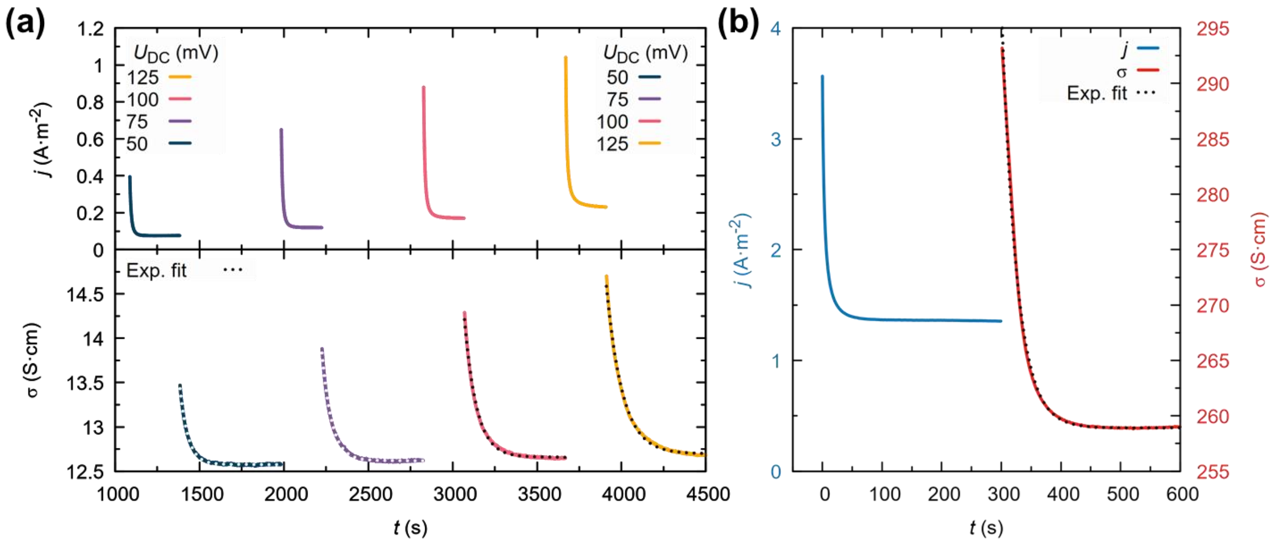


Figure S14: Verification of the applicability of the BCR technique to other perovskite and perovskite-related materials systems: polarization ($j(t)$) and following conductivity relaxation curves ($\sigma(t)$) for (a) several positive voltage steps for a 100 nm thick $La_2NiO_{4+\delta}$ thin film at 360 °C and (b) a single BCR step of 100 mV for a 300 nm thick $YBa_2Cu_3O_{7-\delta}$ thin film at 560 °C in 1000 mbar of $O_2$. Dotted lines are single exponential fitting curves for the conductivity data.

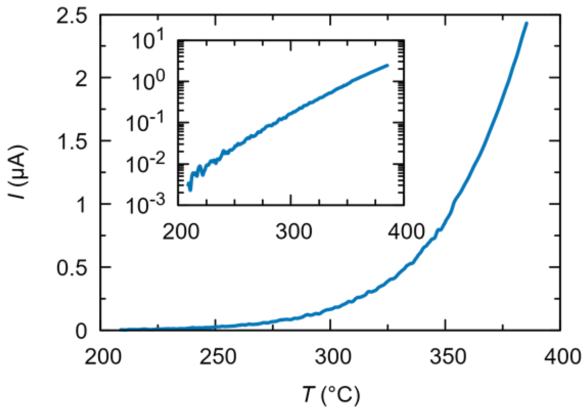


Figure S15: Out-of-plane current, $I(T)$, during cooling while maintaining an applied voltage of 100 mV across the electrochemical cell in polarization geometry. The current decreases exponentially as clearly seen in the semi-log plot in the inset. The strongly reducing current limits BCR measurements to temperatures above around 250-300 °C.

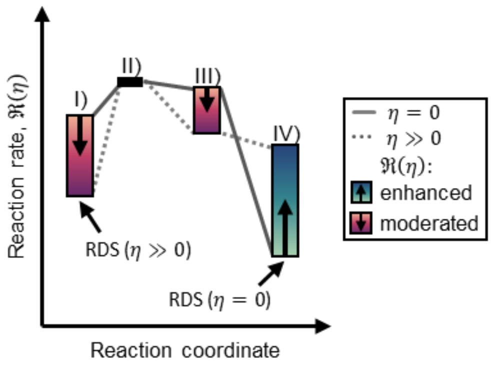


Figure S16: Evolution of reaction rates under applied bias for an exemplary electrochemical cell with (I) oxygen reactions at the counter electrode, oxygen ionic diffusion through (II) the electrolyte and (III) the MIEC thin film and (IV) MIEC surface reactions. In the illustrated example, the RDS changes from step (IV) at 0 bias to step (I) under strong anodic polarizations.
